# Robust Multi-Site ADHD Classification via GraphSAGE-Based Functional Connectivity Modeling from rs-fMRI

**DOI:** 10.3390/bioengineering13050586

**Published:** 2026-05-20

**Authors:** Rabab Bousmaha, Khouloud Meribai, Nardjes Bouchemal, Naila Bouchemal, Galina Ivanova

**Affiliations:** 1 LabRi Laboratory, Ecole Superieure en Informatique, Sidi Bel Abbes 22000, Algeria; k.meribai@esi-sba.dz; 2 LIRE Laboratory, Abdelhamid Mehri Constantine 2 University, Constantine 25000, Algeria; n.bouchemal.dz@ieee.org; 3 LISI Laboratory of Intelligent Systems and Informatics, Mila 43000, Algeria; 4 LyRIDS, ECE Paris, 10 rue Sextius Michel, 75015 Paris, France; naila.bouchemal@ece.fr; 5 Faculty of Electrical Engineering, Electronics and Automation, University of Ruse “Angel Kanchev”, 7017 Ruse, Bulgaria; giivanova@uni-ruse.bg

**Keywords:** ADHD, multi-site data, resting-state fMRI, functional connectivity, PLV, GraphSAGE, graph-based deep learning

## Abstract

Attention Deficit Hyperactivity Disorder (ADHD) is a heterogeneous neurodevelopmental disorder whose diagnosis is mainly based on behavioral assessment and is often delayed due to clinical complexity and limited availability of specialists. Resting-state functional magnetic resonance imaging (rs-fMRI) provides a valuable source of information for supporting automated and objective diagnosis. However, existing studies often do not fully capture the complex interactions of functional connectivity between different brain regions. To address this limitation, this work proposes a graph-based deep learning framework for ADHD classification from rs-fMRI that combines functional connectivity modeling with graph representation learning. The approach used Phase-Locking Value (PLV)-based connectivity estimation and Graph Sample and Aggregate (GraphSAGE) to jointly capture regional brain activity and inter-regional interactions in a scalable and efficient manner. GraphSAGE improves robustness to noise and inter-subject variability by aggregating information from stable local graph neighborhoods. This integration allows the model to learn discriminative connectivity-aware representations while remaining robust to signal variability and adaptable to multi-site data. The proposed framework was evaluated on the publicly available ADHD-200 dataset across multiple acquisition sites as well as on a combined multi-site dataset. The results indicate consistent performance across individual sites and on the combined dataset. The model achieved an Accuracy of 0.89, an AUC of 0.96, and a Specificity of 0.96 on the combined dataset, outperforming several existing methods in this setting. By integrating PLV-based connectivity with GraphSAGE learning, the approach provides an effective and scalable solution for automated ADHD classification from rs-fMRI data, contributing to data-driven approaches for the analysis of neurodevelopmental disorders.

## 1. Introduction

Attention Deficit Hyperactivity Disorder (ADHD) is one of the most prevalent neurodevelopmental disorders in children, and it is mainly characterized by inattention and hyperactivity [1]. This disorder significantly reduces children’s quality of life and places a long-term burden on their families. Early diagnosis is therefore essential, as it helps to minimize the negative effects of ADHD and supports affected children in achieving a safer and more stable transition into adolescence [2,3].

Recent advances in neurotechnology and neural interfaces have enabled more direct analysis of brain activity and improved the understanding of neural mechanisms underlying cognitive and neurodevelopmental disorders, including ADHD [4]. In this context, ADHD diagnosis has attracted increasing attention in the research community, leading to the development of computational models for its automatic detection. Many studies have proposed methods to extract informative features from functional Magnetic Resonance Imaging (fMRI) data. These features are generally categorized into voxel-level and region-level features. Among the most widely used voxel-level features, Regional Homogeneity (ReHo) was introduced by Zang et al. in 2004 [5], while Amplitude of Low-Frequency Fluctuations (ALFF) was proposed by Zang in 2007 and further studied by Yang et al. in 2011, who reported abnormal brain activity in ADHD patients [6,7]. Long et al. employed ReHo and ALFF features extracted from fMRI data to classify early Parkinson’s disease [8], demonstrating their effectiveness in neurological disorder analysis. Although voxel-level features are simple and intuitive to compute, they usually suffer from very high dimensionality, making feature selection a necessary step before classification [9]. Alternatively, region-level features are extracted from predefined brain regions using a hypothesis-driven approach. For example, Eloyan et al. analyzed functional connectivity (FC) between regions of the motor cortex to diagnose ADHD [10]. However, region-level features are often less sensitive to subtle disease-related changes, which may occur within parts of a region or across multiple regions. Consequently, relying solely on simple voxel-level or region-level features may not sufficiently capture disease-specific pathological patterns. This highlights the importance of appropriate feature extraction strategies and careful preprocessing of fMRI data for accurate automated diagnosis of ADHD and related disorders [11,12]. In the field of automated ADHD diagnosis using fMRI data, machine learning (ML) and deep learning (DL) techniques have undergone significant development in recent years. Traditional ML approaches, such as support vector machines (SVM) and random forests, are commonly used to classify hand-crafted features, including functional connectivity measures or signal fluctuation metrics. While these methods generally provide moderate performance, they offer the advantage of better interpretability [12].

More recently, DL approaches have gained increasing attention due to their ability to learn complex spatio-temporal patterns directly from raw neuroimaging data. Convolutional neural networks (2D or 3D CNNs) are widely employed to analyse brain activation maps, including models that integrate fALFF and ReHo features to identify subtle regional abnormalities, particularly in areas such as the prefrontal cortex. More advanced architectures, such as attention-enhanced CNNs or recurrent models (RNN/LSTM), further incorporate temporal dynamics from resting-state fMRI time series, improving sensitivity to ADHD-related variations. In addition, multimodal DL frameworks that combine fMRI with structural or genetic data have been explored and have shown potential to improve diagnostic performance [12].

Recent advances in representation learning, particularly contrastive and self-supervised learning approaches, have further improved the ability of medical AI systems to learn informative features from limited and heterogeneous data, which is highly relevant for neuroimaging applications [13]. Existing deep learning approaches for ADHD classification often struggle to effectively capture complex brain connectivity patterns and to generalize across subjects. Recent studies in applied AI have also highlighted similar challenges in modeling complex biomedical data, particularly regarding generalization and robustness across heterogeneous datasets [14]. To address these limitations, we propose a PLV-based GraphSAGE framework that models rs-fMRI data as graphs and leverages both local and global functional interactions, with PLV quantifying phase synchronization between brain regions to highlight connectivity disruptions characteristic of ADHD. GraphSAGE is particularly suitable due to its inductive learning capability, enabling efficient generalization to unseen subjects while reducing overfitting and computational complexity. Additionally, its sampling strategy facilitates the identification of discriminative connectivity-based biomarkers, supporting accurate and robust classification. The main contributions of this study are as follows:

- A novel graph-based deep learning framework for automated ADHD classification from resting-state fMRI time series.
- Integration of functional connectivity modeling using Phase Locking Value (PLV) to capture inter-regional interactions.
- Use of GraphSAGE layers to learn robust, connectivity-aware node representations that aggregate local graph information.
- Demonstration of the framework’s adaptability and robustness to multi-site neuroimaging data.

The remainder of this paper is structured as follows: Section 2 discusses the related work. Section 3 details the data and preprocessing procedures. Section 4 introduces the proposed method. Section 5 presents the experimental setup and results. Section 6 concludes the paper.

## 2. Related Work

### 2.1. Machine Learning-Based Methods for ADHD Classification

Research on machine learning applications for classifying attention-deficit/hyperactivity disorder (ADHD) has evolved over the years. In 2018, Sen et al. [15] proposed a diagnostic method utilizing structural texture and functional connectivity features extracted from MRI scans in the ADHD-200 dataset, achieving notable accuracy through linear support vector machine (SVM) classifiers. In 2020, Chen et al. [16] introduced a two-step classification approach that leveraged resting-state functional connectivity data and SVM to enhance ADHD detection by focusing on individual connectivity patterns. Also in 2020, Rostami et al. [17] applied decision tree models to behavioral, neuropsychological, and neural markers, enabling differentiation of ADHD subtypes with high precision based on sensory-motor and cognitive parameters. In 2023, Lohani et al. [18] further advanced this line of research by proposing an automated diagnostic framework that combines structural MRI with demographic information. Their approach incorporates feature selection techniques and multiple classifiers to analyze gray matter volume and cortical thickness for ADHD classification. Similarly, in 2023, Lin et al. [19] analyzed neuroimaging data—including white matter microstructure, cortical thickness, and functional connectivity—combined with clinical information from the Adolescents Behavior Cognitive Development (ABCD) dataset to investigate ADHD-related brain changes and evaluate machine learning classifiers for predicting diagnosis. Most recently, in 2024, Zamanzadeh et al. [20] utilized ensemble machine learning models, such as balanced random forest (BRF), XGBoost, and easy ensemble classifier (EEC), on graph-theoretical features extracted from auditory-visual integration networks in the ADHD-200 resting-state fMRI dataset to identify potential ADHD biomarkers. Concurrently, Alsharif et al. [21] (2024) utilized event-related potential data from 221 participants and applied machine learning approaches, reporting an accuracy of 91% using an SVM classifier for ADHD diagnosis. Despite these promising results, traditional machine learning methods remain highly dependent on manual feature engineering. In particular, model performance is strongly influenced by the choice of features, and the same algorithm may yield significantly different outcomes depending on the dataset and the feature extraction strategy.This dependence reduces the generalizability of these models across different datasets. Moreover, traditional machine learning algorithms have limited ability to learn complex relationships among multiple attributes of the data, which restricts their capacity to fully capture the complexity of brain networks [22,23]. Table 1 presents an overview of machine learning approaches for ADHD classification.

### 2.2. Deep Learning Approaches for ADHD and Related Neurodevelopmental Disorders

Research on deep learning applications for classifying neurodevelopmental disorders, such as attention-deficit/hyperactivity disorder (ADHD), has progressed significantly in recent year. In 2017, Zou et al. [12] introduced a three-dimensional convolutional neural network to analyze spatial patterns from functional and structural magnetic resonance imaging data. In 2019, Mao et al. [24] proposed a spatiotemporal method incorporating convolutional and recurrent layers to capture features from functional magnetic resonance imaging data on the ADHD-200 dataset. Also in 2019, Sörös et al. [25] employed independent component analysis and dual regression on resting-state functional magnetic resonance imaging data to examine connectivity patterns in adults with ADHD. In 2020, Li et al. [26] utilized tensor decomposition on resting-state functional magnetic resonance imaging data for brain network features. Zhang et al. [27] developed a separated channel attention convolutional neural network for multi-site resting-state functional magnetic resonance imaging datasets. Riaz et al. [2] presented DeepFMRI, combining convolutional and recurrent neural networks to process raw functional magnetic resonance imaging data on the ADHD-200 dataset. Ji et al. [28] introduced convolutional kernels with element-wise weighting for abnormal brain connectivity patterns. Gao et al. [29] proposed an attention attribute-enhanced network with variational autoencoder for resting-state functional magnetic resonance imaging. Sartipi et al. [30] applied the Stockwell transform to functional magnetic resonance imaging time-series data for frequency-specific features. Chauhan et al. [31] used a deep neural network on functional connectivity coefficients from independent component analysis of resting-state functional magnetic resonance imaging, achieving 95% accuracy. In 2021, Wang et al. [32] designed a three-dimensional multiscale convolutional neural network with attention for magnetic resonance imaging data. Qiang et al. [33] developed a deep variational autoencoder for mapping functional brain networks. Khullar et al. [34] employed two-dimensional convolutional neural networks and hybrid convolutional neural network-long short-term memory models on resting-state functional magnetic resonance imaging data. De Silva et al. [35] integrated seed-based correlation, fractional amplitude of low-frequency fluctuations, and regional homogeneity features with convolutional neural networks, attaining accuracies of 84% to 86%. In 2022, Chen et al. [36] introduced a multi-filter convolutional neural network for brain connectomes. Liu et al. [37] advanced nested residual convolutional denoising autoencoders for spatio-temporal features from functional magnetic resonance imaging data. Qiang et al. [38] presented a resting-state temporal templates method using spatiotemporal attention autoencoders, exceeding 93% accuracy. Simeon et al. [39] investigated Riemannian geometry for harmonizing multisite ADHD data and preserving functional connectivity patterns. Ke et al. [40] proposed a deep learning approach with self-attention factorization for functional connectivity patterns. Qin et al. [41] developed the Trans3D-ensemble model, fusing spatio-temporal functional magnetic resonance imaging features with phenotypic data using three-dimensional convolutional neural networks, transformers, and random forests, achieving 74.5% accuracy. In 2023, Hsieh et al. [42] introduced a data-driven seed-correlation method on resting-state functional magnetic resonance imaging, reaching 83.24% accuracy on the ADHD-200 NYU dataset. Also in 2023, Chen et al. [3] proposed an attention auto-encoding neural network combined with biomarker detection for ADHD classification. In 2024, Mengi et al. [43] proposed an unsupervised multi-source domain adaptation network for structural magnetic resonance imaging and functional magnetic resonance imaging data. Firouzi et al. [44] presented Skip-Vote-Net, leveraging dynamic functional connectivity matrices from resting-state functional magnetic resonance imaging for ADHD subtypes using majority voting. In 2025, Xue et al. [45] introduced topological manifold learning on resting-state functional magnetic resonance imaging data for ADHD diagnostics and severity assessment. Deep learning models typically represent brain data as two-or three-dimensional matrices, which can lead to the loss of important structural information due to the complex and critical connections between different brain regions. In contrast, Graph Neural Networks (GNNs) are specifically designed for graph-structured data, allowing them to capture these complex dependencies by aggregating and propagating information across nodes and their neighbors, thus effectively modeling the intricate relationships within brain networks [46]. Table 2 provides a summary of representative deep learning approaches for ADHD classification using fMRI data.

### 2.3. Graph-Based Approaches for ADHD Classification

Recent studies have advanced graph-based approaches for ADHD classification using rs-fMRI by incorporating dynamic and higher-order brain network representations. Early graph-based models include the population-based graph convolutional network proposed by Parisot et al. [47], which models subjects as nodes and encodes inter-subject similarities using phenotypic information, as well as BrainGNN introduced by Li et al. [48], an interpretable brain graph neural network that represents each subject as an individual brain graph with regions of interest as nodes and functional connections as edges.

Extending these initial graph-based approaches, Zhao et al. [49] proposed a dynamic graph convolutional neural network to analyze time-varying functional brain networks derived from resting-state fMRI. Their model captured temporal variations in functional connectivity, leading to improved classification performance and the identification of abnormal connections associated with clinical symptoms. Following this, Zhang et al. [50] introduced an adversarial graph contrastive learning (A-GCL) framework. Their method constructs dynamic graphs and applies contrastive learning to enhance graph representations. This approach effectively handles topological disturbances in brain networks, learns robust features, and achieved an accuracy of 70.92% for ADHD classification. Subsequently, Hu et al. [51] applied a graph convolutional network (GCN) to investigate abnormal functional connectivity patterns in individuals with ADHD. Their results showed that graph-based learning effectively distinguishes ADHD subjects from typically developing controls and highlights key brain regions involved in attention and cognitive control networks. In 2025, Wu et al. [52] introduced the HAGCN framework, a hybrid-order brain network-based graph convolutional model with multi-head attention. This approach captures both local and higher-order topological properties of brain networks and was applied to the classification of brain disorders, including ADHD.

Table 3 summarizes the main graph-based approaches for ADHD classification using rs-fMRI.

## 3. Data and Preprocessing

### 3.1. Dataset

In this study, we use resting-state fMRI time-series data from the ADHD-200 consortium, preprocessed using the Athena pipeline, which combines the AFNI and FSL toolkits [53]. The ADHD-200 dataset includes structural and resting-state fMRI data collected at eight different imaging sites, along with phenotypic and diagnostic information distinguishing individuals with ADHD from typically developing controls [54]. For our experiments, we selected data from the NYU, KKI, Peking, OHSU, and NeuroIMAGE sites, and we also created a combined dataset by adding data from the WashU and Pittsburgh sites. This choice allows us to test the performance of our models across different acquisition sites and populations, and to examine whether the results remain consistent beyond a single site. To provide a clear overview of the dataset, Table 4 presents the number of participants per site and the distribution between ADHD and typically developing control groups, highlighting differences across sites.

### 3.2. Preprocessing

The preprocessing steps used in this study rely on the Athena pipeline from the ADHD-200 Preprocessed project, developed by the Neuro Bureau [53]. The Athena pipeline converts the raw 4D fMRI volumes into subject-level time series through the following steps:

- Initial preprocessing of functional images: Slice timing correction, rigid-body motion correction, and co-registration of functional scans to each subject’s structural image.
- Spatial normalization: Non-linear registration of structural images to MNI152 space, with functional scans resampled accordingly.
- Nuisance regression: Removal of confounding signals, including six motion parameters, mean white matter, and cerebrospinal fluid signals.
- Temporal filtering: Band-pass filtering (typically 0.009–0.08 Hz) to retain physiologically meaningful fluctuations.
- Spatial smoothing: Gaussian smoothing applied to improve signal-to-noise ratio.
- Extraction of ROI-level time series: Preprocessed functional data are parcellated into regions of interest (ROIs) using the Automated Anatomical Labeling (AAL) atlas. For each subject and each ROI, the mean BOLD signal is computed across voxels, producing a 2D matrix (time points × ROIs).

**Figure 1:**
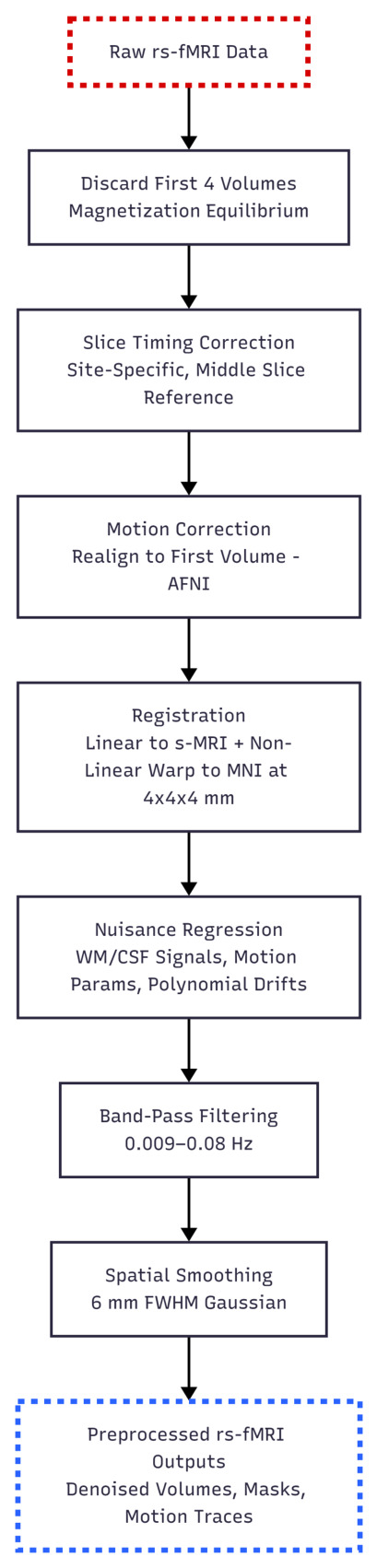
Athena Pipeline for rs-fMRI Preprocessing.

As a result, for every subject, the data consist of a tabular time series file where rows correspond to time points and columns correspond to ROIs defined in the AAL atlas. Figure 1 presents the main preprocessing workflow of the Athena pipeline for resting-state fMRI data.

To increase the diversity of the training data and enhance model generalization, data augmentation techniques were applied directly to the ROI-level time series. The augmentation was performed after splitting the dataset for cross-validation and was restricted to the training folds only. Specifically:

- Additive Gaussian noise: A small amount of random noise, sampled from a normal distribution $N(0,\sigma^{2})$ with σ=0.01, was added to the time series:
  (1) $$\tilde{X}=X+\epsilon ,\;\epsilon ∼N\left(0,\sigma^{2}\right).$$
  This step simulated variability in measurement noise.
- Temporal shifting: The time series were shifted using a circular operation (wrap-around) with a fixed offset (shift = 5 time points), implemented via a synchronous shift across all ROIs (using np.roll). This operation preserves the relative temporal structure between regions while generating alternative aligned versions of the same signals. Although this transformation does not modify phase relationships and therefore does not affect PLV-based connectivity, it introduces slight variations in the raw temporal inputs provided to the model.

These augmentations were applied independently, producing additional synthetic samples while preserving the general structure of the original signals.

## 4. Proposed Method

This work proposes a graph-based framework for the analysis of resting-state fMRI time series, aiming to achieve accurate and robust classification. To this end, the Automated Anatomical Labeling (AAL) atlas was used to define regions of interest (ROIs), such that each node in the graph corresponds to a meaningful anatomical brain region. From the time series associated with each ROI, a set of statistical and spectral features was extracted to characterise regional brain activity. Functional interactions between ROIs were then quantified using the Phase Locking Value (PLV), a phase-based connectivity measure that captures synchronisation between brain signals. These connectivity measures were used to construct a subject-level brain graph, where nodes represent ROIs and edges represent functional connections. Finally, the resulting graphs were classified into ADHD and control groups using GraphSAGE, a graph neural network model that learns node representations by aggregating information from local neighbourhoods. This approach allows the model to capture both regional properties and inter-regional interactions, while remaining scalable and efficient. The following subsections detail each stage of the proposed pipeline, including feature extraction, functional connectivity estimation, graph construction, GraphSAGE model design, training procedure, and evaluation.

### 4.1. Feature Extraction

Each subject’s resting-state fMRI time series was represented as a matrix of size T×N, where *T* denotes the number of time points and *N* corresponds to the number of regions of interest (ROIs) defined by the AAL atlas (N=116). To capture relevant temporal and spectral characteristics of each ROI signal, a compact set of 13 features was extracted, describing both statistical and dynamic properties of the BOLD signal. These features were computed independently for each ROI and concatenated to form the node-level feature matrix for each subject.

#### 4.1.1. Statistical Descriptors

For each ROI time series x(t), the mean μ and standard deviation σ were used to quantify the average activation level and signal variability:

(2) $$\mu =\frac{1}{T}\sum_{t=1}^{T}x\left(t\right),\;\sigma =\sqrt{\frac{1}{T}\sum_{t=1}^{T}{\left(x(t)-\mu \right)}^{2}}.$$

The skewness (skew) and kurtosis (kurt) were computed to describe the shape of the signal amplitude distribution:

(3) $$\mathrm{skew}=\frac{E\left[{\left(x-\mu \right)}^{3}\right]}{\sigma^{3}},\;\mathrm{kurt}=\frac{E\left[{\left(x-\mu \right)}^{4}\right]}{\sigma^{4}}.$$

These higher-order statistical moments have been shown to capture abnormal temporal fluctuations in resting-state networks associated with neuropsychiatric conditions [55,56].

#### 4.1.2. Energy Measures

Two amplitude-based energy measures were considered: the root-mean-square (RMS) amplitude and the total signal energy. The RMS amplitude is defined as

(4) $$\mathrm{RMS}=\sqrt{\frac{1}{T}\sum_{t=1}^{T}x{\left(t\right)}^{2}}.$$

while the total energy is given by

(5) $$E=\sum_{t=1}^{T}x{\left(t\right)}^{2}.$$

These measures quantify the power of BOLD signal fluctuations and have been previously used to identify hypoactivation patterns in ADHD [57].

#### 4.1.3. Temporal Dynamics and Signal Morphology

To capture signal complexity, we compute the number of zero crossings (ZC) as

(6) $$\mathrm{ZC}=\sum_{t=2}^{T}I\left[x(t-1)\cdot x(t)<0\right].$$

which measures how frequently the signal changes sign and reflects oscillatory irregula- rity [58]. We also estimate short-term temporal dependency using the lag-1 autocorrelation, defined as the Pearson correlation between consecutive samples:

(7) $$\rho_{1}=\frac{\mathrm{cov}\left(x_{1:T-1},\;x_{2:T}\right)}{\sigma^{2}}.$$

where $\sigma^{2}$ denotes the signal variance. This measure describes the persistence of neural fluctuations across adjacent time points [59].

#### 4.1.4. Spectral Features

Using Welch’s method [60], we computed the power spectral density (PSD) P(f) with a sampling frequency of 0.5 Hz (corresponding to a repetition time TR≈2 s) [53]. Two features were derived from the PSD:

1. **Spectral entropy** [61]: a measure of frequency-domain irregularity,
  (8) $$H_{s}=-\sum_{f}p\left(f\right)\;\log p\left(f\right),\;p\left(f\right)=\frac{P(f)}{\sum_{f}P\left(f\right)}.$$
  where higher values indicate more distributed spectral power.
2. **Mean frequency** $\bar{f}$, expressing the centre of spectral mass,
  (9) $$\bar{f}=\frac{\sum_{f}f\;p\left(f\right)}{\sum_{f}p\left(f\right)}.$$

Spectral features have been widely used to characterise frequency-dependent dysconnectivity in ADHD and other developmental disorders [62].

#### 4.1.5. Time-Frequency (Wavelet) Features

To characterise transient changes across multiple temporal scales, we applied a discrete wavelet transform (DWT) using Daubechies-4 (db4) wavelets up to level 3. From the detail coefficients at the final level ($d_{3}$), we computed their energy:

(10) $$E_{w}=\sum_{t}d_{3}{\left(t\right)}^{2}.$$

which captures the contribution of high-frequency components to the overall signal variance [63].

#### 4.1.6. Robust Amplitude Statistics

To obtain robust measures of signal amplitude, we computed the 25th and 75th percentiles of each ROI’s signal distribution ($Q_{1}$ and $Q_{3}$), capturing the lower and upper bounds of activity while reducing sensitivity to outliers.

#### 4.1.7. Feature Structure

Each ROI is represented by a 13-dimensional feature vector combining temporal, spectral, and statistical information. All features were normalized using z-score standardization across the training set. This produces a subject-level feature matrix of size N×13, where N=116 corresponds to the ROIs defined by the AAL atlas.

#### 4.1.8. Overview of Extracted Node-Level Features

Table 5 provides an overview of the features extracted for each ROI. The feature set includes:

This feature design provides a compact yet informative representation of regional BOLD dynamics, while remaining computationally efficient. In contrast to dictionary learning or ICA-based representations [64], the atlas-based feature extraction preserves anatomical meaning at the node level, supporting subsequent analyses.

### 4.2. Functional Connectivity

After extracting node-level features, we estimated pairwise functional connectivity between brain regions using a phase-based synchrony measure. Specifically, we employed the Phase Locking Value (PLV), which assesses the consistency of phase differences between two BOLD signals over time. PLV is widely used in EEG and fMRI connectivity studies because it detects stable phase relationships while remaining insensitive to variations in signal amplitude, thereby offering greater robustness to noise and scaling effects compared to correlation-based measures [64,65,66].

#### 4.2.1. Phase Extraction

For each region-of-interest (ROI) time series x(t), the instantaneous phase ϕ(t) was derived from the analytic signal obtained through the Hilbert transform:

(11) $$\tilde{x}\left(t\right)=x\left(t\right)+i\;H\left\{x\left(t\right)\right\},\;\phi \left(t\right)=\arg\left(\tilde{x}\left(t\right)\right).$$

where H{·} represents the Hilbert transform. This transformation applies a $90^{\circ }$ phase shift to each frequency component, enabling the calculation of an instantaneous phase for narrowband BOLD oscillations [64,67]. Although the Hilbert transform assumes narrowband signals for theoretically well-defined phase estimation [68], its use remains common in resting-state fMRI phase synchronization studies [67,69,70]. In this work, the 0.009–0.08 Hz band-pass filter spans a relatively broad frequency range, which does not strictly satisfy the narrowband condition. Therefore, the resulting phase estimates should be interpreted with caution as a methodological limitation.

#### 4.2.2. Phase Synchrony (PLV) Computation

For any pair of regions of interest (ROIs) *p* and *q*, with respective instantaneous phases $\phi_{p}\left(t\right)$ and $\phi_{q}\left(t\right)$, the Phase Locking Value (PLV) was calculated as follows:

(12) $${\mathrm{PLV}}_{pq}=\left|\frac{1}{T}\sum_{t=1}^{T}e^{\;i\left(\phi_{p}\left(t\right)-\phi_{q}\left(t\right)\right)}\right|.$$

where *T* is the number of time points. PLV values range from 0 (no consistent phase relationship) to 1 (perfect phase locking). Thus, PLV provides a measure of how consistently the phases of the two signals remain aligned over time, serving as an index of functional coupling strength between brain regions. In practice, we computed the PLV for every pair of ROIs using each participant’s time series. This resulted in a symmetric N×N connectivity matrix, where N=116 (corresponding to the Automated Anatomical Labeling atlas). The diagonal elements were set to 1, while off-diagonal elements represented the degree of phase synchrony between distinct regions. One such PLV matrix was generated for each participant.

#### 4.2.3. Connectivity Thresholding and Adjacency Construction

To construct graphs from the dense Phase Locking Value (PLV) matrices, a percentile-based thresholding method was applied to retain only the strongest functional connections. Within each outer cross-validation fold, a threshold τ was computed exclusively from the distribution of PLV values of the training data, excluding validation and test sets, with diagonal elements removed. An edge between nodes *p* and *q* was retained when ${\mathrm{PLV}}_{pq}\geq \tau$. In this study, τ was set to the 80th percentile of the PLV distribution derived from the training data, thereby preserving the strongest 20% of functional connections. The same threshold was then applied to both the validation subset and the held-out test fold within each split. This strategy ensures consistent graph density while preventing any information leakage from non-training data [64,71,72].

### 4.3. Graph Construction

After computing the functional connectivity matrices, each subject was represented set of functional connections (edges), and *A* the corresponding adjacency matrix obtained from the thresholded Phase Locking Value (PLV) matrix.

#### 4.3.1. Adjacency Matrix

For each subject, the PLV matrix was binarized using a global percentile-based threshold. Specifically, entries with ${\mathrm{PLV}}_{ij}\geq \tau$ were assigned a value of 1, while all other entries were set to 0:

(13) $$A_{ij}=\left\{\begin{array}{cc} 1, & \mathrm{if}\;{\mathrm{PLV}}_{ij}\geq \tau ,\\ 0, & \mathrm{otherwise}. \end{array}\right.$$

Self-connections were explicitly removed by setting $A_{ii}=0$. This procedure resulted in sparse, undirected binary graphs that capture the strongest functional synchronization patterns between AAL-defined brain regions. Each graph consists of N=116 nodes, while the number of edges varies according to the threshold value τ.

#### 4.3.2. Graph Metrics

To enrich node representations with topological information, two fundamental graph-theoretic measures were computed from the adjacency matrices using the NetworkX library [73]:

- **Node degree**: the number of direct connections of node *i*,
  (14) $$k_{i}=\sum_{j}A_{ij}.$$
  reflecting its local connectivity. Nodes with higher degrees are typically considered network hubs involved in information integration [74].
- **Clustering coefficient**: quantifies the tendency of a node’s neighbours to be interconnected,
  (15) $$C_{i}=\frac{2T_{i}}{k_{i}\left(k_{i}-1\right)}.$$
  where $T_{i}$ is the number of triangles involving node *i*. This measure captures local segregation and network modularity, which have been shown to differ between ADHD and control groups [75].

These graph metrics were computed for each node and concatenated with the region-of-interest (ROI)-based feature vectors obtained from the time-series analysis. Consequently, the resulting feature matrix for each subject combines two complementary sources of information: (1) the local temporal and spectral dynamics of each brain region, and (2) the topological properties of each region within the functional network.

Incorporating graph-derived features enables the model to learn from both the intrinsic activity of individual brain regions and their inter-regional topological organization. The node degree highlights central regions that function as network hubs, while the clustering coefficient captures community-like structures that may reflect local network integrity. This combined representation has been shown to enhance graph-based classification performance and improve interpretability in neuroimaging studies [72,76].

**Figure 2:**
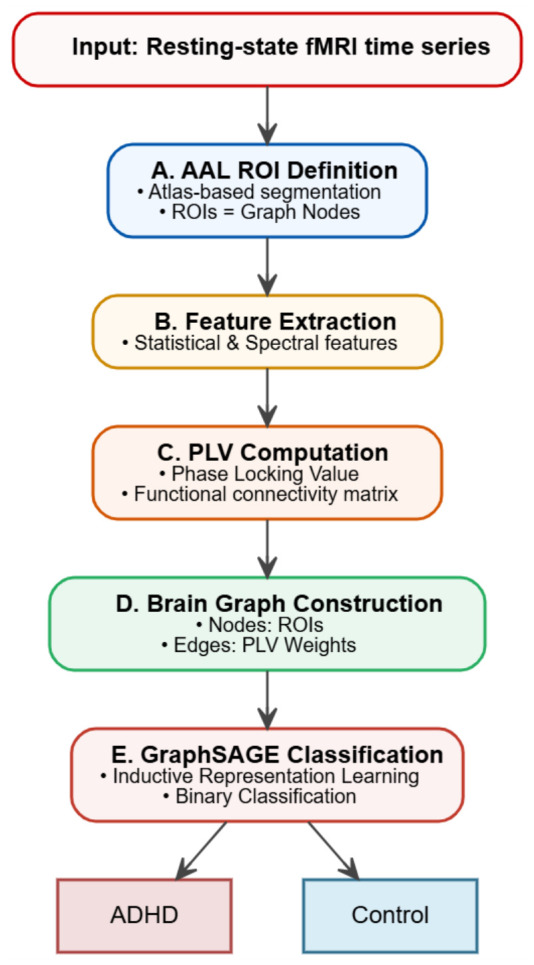
General workflow of the proposed graph-based approach for resting-state fMRI analysis and ADHD classification.

In summary, this step converts each subject’s functional connectivity data into a brain graph with anatomically defined nodes, functionally meaningful edges, and enriched node-level features. These graphs serve as input to the Graph SAmple and aggreGatE (GraphSAGE) classifier described in the following subsection. Figure 2 presents an overview of the proposed graph-based framework for resting-state fMRI analysis and ADHD classification.

### 4.4. Proposed Model

The final stage of the pipeline involves classifying each subject’s brain graph using a GraphSAGE model. GraphSAGE extends standard Graph Convolutional Networks (GCNs) by learning aggregation functions that combine information from a node’s neighbors. This approach is particularly valuable for fMRI connectivity data, where not all brain regions or connections contribute equally to the discriminative patterns of neural activity. By aggregating the most relevant neighborhood features, GraphSAGE can generate meaningful node embeddings while remaining scalable to large and complex brain graphs.

#### 4.4.1. Graph SAmple and AggreGatE (GraphSAGE)

GraphSAGE, as introduced in [77], is a fundamental inductive graph neural network (GNN) framework designed to learn node representations for large-scale and evolving graphs. Unlike transductive methods, GraphSAGE learns parametric aggregation functions that combine a node’s features with information from its local neighborhood.

GraphSAGE supports several aggregation strategies, including mean aggregation, max-pooling, and LSTM-based aggregation. In this work, we use mean aggregation, which computes the element-wise average of the sampled neighbor features and combines it with the node’s own representation through a learnable linear transformation and non-linear activation function.

At layer *l*, the node representation is updated as follows:

(16) $$h_{v}^{\left(l+1\right)}=\sigma \left(W_{1}h_{v}^{\left(l\right)}+W_{2}\cdot \frac{1}{\left|N(v)\right|}\sum_{u\in N(v)}h_{u}^{\left(l\right)}\right),$$

where N(v) is the set of sampled neighbors, $W_{1}$ and $W_{2}$ are learnable weight matrices, and σ is a non-linear activation function.

Mean aggregation can be seen as an inductive generalization of Graph Convolutional Networks (GCNs). It balances expressiveness, computational cost, and generalization ability, making it a widely used baseline in inductive graph learning [77].

#### 4.4.2. Input Representation

Each subject is represented as a graph G=(V,E,X), where V denotes the set of nodes corresponding to AAL regions, E the set of functional edges obtained through PLV thresholding, and $X\in R^{N\times F}$ the node feature matrix.

Each node feature vector integrates two types of information:

1. Statistical and spectral descriptors extracted from the regional BOLD time series;
2. Graph-theoretic measures, specifically the degree and clustering coefficient, computed from the connectivity topology.

All features were normalized based on the training data within each cross-validation fold to prevent data leakage.

#### 4.4.3. Model Architecture

The proposed GraphSAGE-based model consists of three GraphSAGE convolutional layers followed by a fully connected classifier, with Layer Normalization for improved training stability:

1. **SAGEConv-1**: Projects input features into 64 hidden dimensions, followed by LayerNorm, ReLU activation, and dropout (0.3). This layer aggregates neighbor information using mean aggregation via the GraphSAGE operation:
  (17) $$h_{i}^{\prime }=\sigma \left(W\cdot \mathrm{CONCAT}\left(h_{i},\;{\mathrm{MEAN}}_{j\in N(i)}\left\{h_{j}\right\}\right)\right)$$
  where N(i) denotes the neighbors of node *i*, and σ non-linear activation function.
2. **LayerNorm-1**: Applies layer normalization to intermediate embeddings to stabi- lize training:
  (18) $$\mathrm{LN}\left(h\right)=\gamma \cdot \frac{h-\mu }{\sqrt{\sigma^{2}+\epsilon }}+\beta .$$
3. **SAGEConv-2**: A second SAGEConv layer that refines the node embeddings, followed by LayerNorm, ReLU, and dropout (0.3).
4. **SAGEConv-3**: A third SAGEConv layer that further transforms node embeddings, followed by LayerNorm and ReLU.
5. **Global Mean Pooling**: Aggregates all node embeddings into a single graph- level vector:
  (19) $$h_{G}=\frac{1}{\left|V\right|}\sum_{i\in V}h_{i}.$$
6. **Fully Connected Classifier**: Two dense layers (64 → 32 → 2) with ReLU activations and dropout (0.3) output the final ADHD/TDC prediction via softmax.

**Figure 3:**
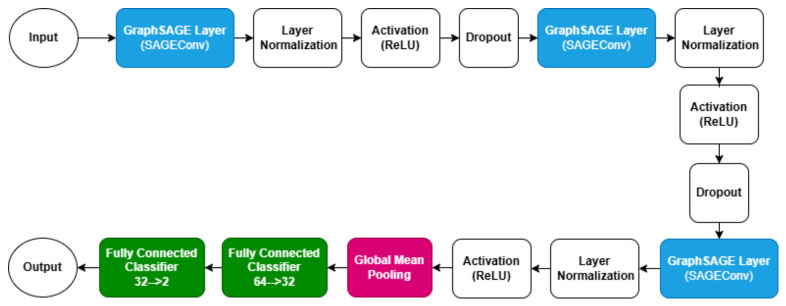
Architecture of the proposed model.

This architecture captures both local neighborhood interactions through SAGEConv aggregation and global graph-level patterns via pooling. Figure 3 illustrates the architecture of the proposed model. Algorithm 1 outlines the key steps of our graph-based approach for classifying ADHD from resting-state fMRI data, including ROI definition, feature extraction, connectivity estimation, graph construction, and GraphSAGE classification. The complete source code of the implementation is provided in the Supplementary Materials (File S1).
**Algorithm 1** Pseudocode for the Proposed Graph-Based Framework for ADHD Classification from Resting-State fMRI  1:**Input:**  2:   - time_series_fMRI: fMRI time series (T×116)  3:   - labels: Class labels (ADHD/Control)  4:**Output:**  5:   - prediction: Classification (ADHD/Control)  6:// A: Define ROIs  7:ROIs← AAL atlas (116 regions)  8:// B: Extract node features  9:**for** each ROI *i* **do**10:    signal←time_series_fMRI[:,i]11:    Compute 13 features: stats (mean, std, skew, kurt), energy (RMS, total), temporal (ZC, autocorr), spectral (entropy, mean freq), wavelet energy, percentiles (Q1, Q3)12:**end for**13:Normalize features14:// C: Compute functional connectivity (PLV)15:**for** each ROI pair (p,q) **do**16:    Extract phases via Hilbert transform17:    $PLV_{pq}\leftarrow \left|\frac{1}{T}\sum_{t=1}^{T}e^{i(\phi_{p}\left(t\right)-\phi_{q}\left(t\right))}\right|$18:**end for**19:Threshold PLV matrix at 80th percentile to get binary adjacency20:// D: Construct graph21:Create graph with nodes (ROIs + features) and edges (from adjacency)22:**for** each node **do**23:    Append degree and clustering coefficient to features24:**end for**25:// E: Classify26:Train GraphSAGE on graphs and labels27:prediction← GraphSAGE inference on graph28:**return** prediction

## 5. Experiments and Results

In this section, we assess the performance of our proposed method for ADHD classification using resting-state fMRI data, and we compare our results with those from state-of-the-art approaches in the literature.

### 5.1. Experimental Setting

All experiments were conducted using the cloud-based computational environment provided by Kaggle. The experiments were executed on a GPU-enabled notebook equipped with dual NVIDIA Tesla T4 GPUs (2 × 16 GB VRAM), 31 GB of system RAM, and a 4-core Intel Xeon CPU (2.00 GHz). The training process leveraged both GPUs to accelerate model optimization and improve computational efficiency. The proposed model was implemented in Python 3.12.13 using the PyTorch 2.10.0 deep learning framework, while graph-based operations were performed using PyTorch Geometric. Data preprocessing and performance evaluation were carried out using standard scientific computing libraries, including NumPy and Scikit-learn. Experiments were conducted on the ADHD-200 dataset, which comprises resting-state fMRI data collected from multiple imaging sites, along with corresponding phenotypic and diagnostic labels (ADHD vs. typically developing controls). The dataset exhibits a noticeable class imbalance, with the ADHD class representing the minority. All experiments were performed at the subject level, where each subject was represented by a graph with associated node-level features. We employed a five-fold cross-validation scheme (*k* = 5) to ensure balanced label distribution across folds. In each outer iteration, the model was trained on four folds and evaluated on the remaining fold, used as an independent test set. Within the training portion (i.e., the four folds), a single stratified split was performed to further divide the data into training and validation subsets, using three folds for training and one fold for validation. This validation set was used for model selection, while the test set remained completely unseen. This design does not correspond to a full nested cross-validation procedure, as only a single validation split is used within each outer fold, but it ensures an efficient model selection process while maintaining a strict separation between training and test data. Node-level features from the training subjects were used to fit a standard scaler, ensuring that normalization parameters were learned exclusively from the training data and subsequently applied to the test set. Model weights and classification thresholds were re-estimated independently for each fold.

The main performance metrics included accuracy, precision, recall, Specificity, F1-score, and area under the ROC curve (AUC). This procedure allowed for a reliable assessment of model performance while preserving class distribution and avoiding data leakage between training and test sets.

#### 5.1.1. Optimisation and Regularisation

The model was trained using the Adam optimiser with an initial learning rate of $1\times 10^{-3}$ and a weight decay of $1\times 10^{-5}$ and $1\times 10^{-3}$ for the individual sites. Training was conducted for up to 100 epochs per fold for each of the five individual sites and up to 200 epochs for the combined dataset. Early stopping was applied with a patience of 15 epochs for the individual sites and 30 epochs for the combined dataset to prevent overfitting, particularly in smaller site-specific datasets. This adaptive training strategy was designed to balance convergence and generalization according to dataset size, reducing overfitting risk in smaller site-specific datasets while allowing deeper optimization on the larger combined dataset.

To stabilise training, gradient clipping with a maximum norm of 1.0 was applied after each backward pass. In addition, a ReduceLROnPlateau scheduler reduced the learning rate by a factor of 0.5 when validation precision (or F1-score) did not improve for ten epochs.

#### 5.1.2. Class Imbalance Handling

To address class imbalance, class weights were computed for each fold using the balanced strategy of the compute_class_weight function from scikit-learn. The weight assigned to the minority class was further scaled by a factor of 3.0 to mitigate bias toward the majority class. Model training was performed using a weighted cross-entropy loss function defined as follows:

(20) $$L=-\sum_{c\in \{0,1\}}w_{c}\;y_{c}\log\left({\hat{y}}_{c}\right),$$

where $w_{c}$ denotes the class weight and ${\hat{y}}_{c}$ the predicted probability for class *c*.

#### 5.1.3. Validation Strategy and Adaptive Threshold Optimisation

After each training epoch, model predictions on the validation set were evaluated using accuracy, precision, recall, specificity, F1-score, and AUC. Given that clinical screening tasks prioritise sensitivity (recall) over specificity—particularly for the positive (ADHD) class—the classification threshold was not fixed at the conventional value of 0.5. Instead, an adaptive threshold optimisation strategy was employed.

For each validation fold, the optimal probability threshold $\theta^{*}$ was determined using a grid search over the interval [0.01,0.99], with the constraint that recall remains above a minimum acceptable level to ensure clinically meaningful sensitivity. Specifically, the selected threshold maximizes precision while enforcing a recall of at least 0.75:

(21) $$\theta^{*}=\arg\max_{\theta }\;\mathrm{Precision}\left(\theta \right)\;s.t.\;\mathrm{Recall}\left(\theta \right)\geq r_{\min}.$$

Rather than aiming to maximize recall, this approach ensures that sensitivity does not fall below a predefined clinical requirement. Within this constraint, the threshold is chosen to reduce false positives as much as possible. In practice, this leads to more consistent behavior across validation folds and helps maintain stable performance across different sites.

#### 5.1.4. Evaluation Metrics and Performance Aggregation

Model performance was assessed using accuracy, precision, recall, specificity, F1-score, and area under the ROC curve (AUC). Final results were reported as the mean and standard deviation across the five folds:

(22) $$\begin{array}{cc} {\mathrm{Metric}}_{\mathrm{avg}} & =\frac{1}{K}\sum_{k=1}^{K}{\mathrm{Metric}}_{k}, \end{array}$$

(23) $$\begin{array}{cc} \sigma & =\sqrt{\frac{1}{K}\sum_{k=1}^{K}{\left({\mathrm{Metric}}_{k}-{\mathrm{Metric}}_{\mathrm{avg}}\right)}^{2}}. \end{array}$$

where *K* denotes the number of folds in the cross-validation procedure, ${\mathrm{Metric}}_{k}$ represents the value of the considered evaluation metric (e.g., accuracy, precision, recall, F1-score, or AUC) obtained on the *k*-th fold, ${\mathrm{Metric}}_{\mathrm{avg}}$ corresponds to the average performance across all folds, and σ denotes the corresponding standard deviation, reflecting the variability of the model performance across different data splits.

Table 6 presents a summary of the training settings used in this study.

### 5.2. Results

**Figure 4:**
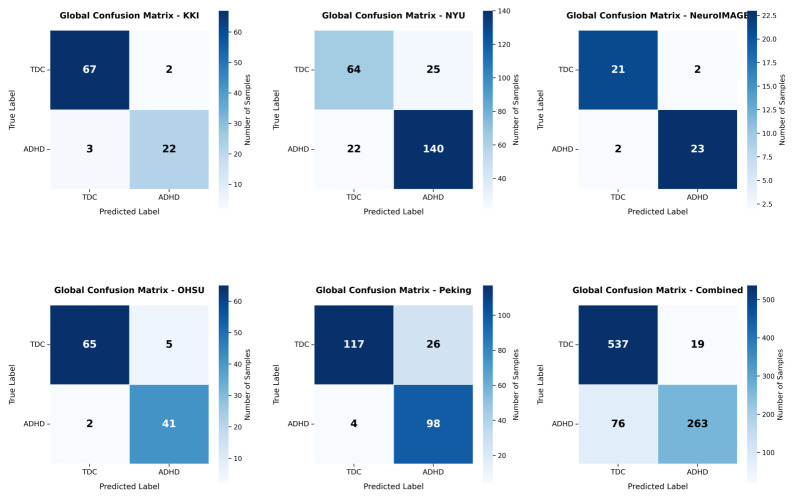
Confusion matrices of the GraphSAGE classifier for each site.

**Figure 5:**
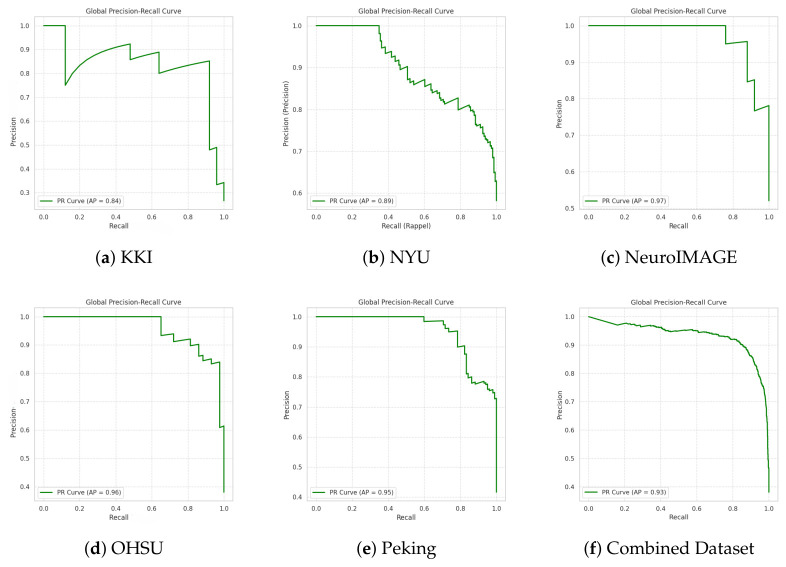
Precision–Recall (PR) curves for each site.

**Figure 6:**
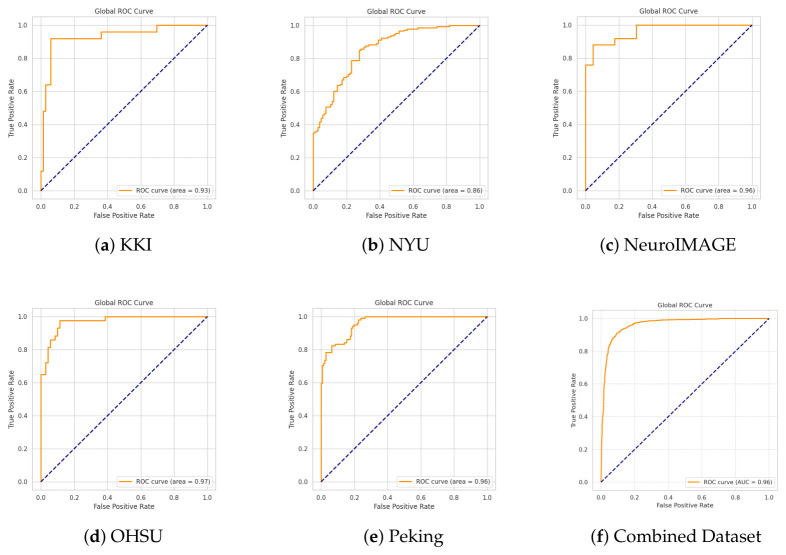
Receiver Operating Characteristic (ROC) curves for each site.

Model performance was evaluated independently at each imaging site to assess the robustness and generalisability of the proposed PLV-GraphSAGE-based ADHD detection across heterogeneous acquisition protocols. Performance was reported using accuracy, precision, recall (sensitivity), specificity, F1-score, and area under the ROC curve (AUC). Mean and standard deviation across sites were used to summarise stability and discriminative performance. These metrics measure the ability of the model to distinguish ADHD from typically developing control (TDC) subjects based on graph representations derived from ROI-level time-series data. Precision and recall were threshold-optimised to ensure a minimum sensitivity of 0.75. To further illustrate classification performance, Figure 4 shows the confusion matrices for each imaging site, where diagonal entries indicate correct predictions and off-diagonal elements represent misclassifications between ADHD and TDC classes. Receiver operating characteristic (ROC) and precision–recall (PR) curves were also used to evaluate the trade-off between sensitivity and specificity across different decision thresholds (Figure 5 and Figure 6).

### 5.3. Discussion

Table 7 reports the classification performance of the proposed PLV-GraphSAGE framework across individual ADHD-200 acquisition sites and the combined dataset, demonstrating robust and stable discriminative capability despite inter-site heterogeneity. The model achieves an average accuracy of 0.900 and a mean ROC-AUC of 0.908, indicating strong separation between ADHD and typically developing subjects and confirming reliable probabilistic ranking across operating points. Precision remains relatively high across sites (average ≈ 0.905), while recall averages 0.894, reflecting effective detection of ADHD cases. The mean F1-score of 0.891 further confirms a balanced trade-off between precision and recall, ensuring that performance is not driven by a single metric.

In addition, the average specificity reaches 0.886, indicating that the model effectively identifies typically developing controls while maintaining controlled false-positive rates. For instance, KKI and the Combined Dataset show particularly high specificity values (0.971 and 0.965, respectively), demonstrating strong ability to correctly classify non-ADHD subjects. In contrast, sites such as Peking exhibit slightly lower specificity (0.818), which is consistent with its sensitivity-oriented behavior characterized by very high recall (0.961). This reflects different operating balances rather than instability. Site-specific variations are expected in multi-site neuroimaging studies due to differences in acquisition protocols, demographic characteristics, and preprocessing pipelines. For example, KKI achieves high accuracy (0.947) and strong F1-score (0.897) with high specificity (0.971), reflecting well-balanced classification. OHSU and NeuroIMAGE also demonstrate stable and competitive performance across all metrics. Importantly, the Combined Dataset maintains competitive performance (accuracy = 0.898 ± 0.022, recall = 0.787 ± 0.044, specificity = 0.965 ± 0.017, F1 = 0.852 ± 0.012, AUC = 0.958 ± 0.012), confirming good generalization under heterogeneous conditions. Although recall in the Combined Dataset is more moderate, specificity remains very high, indicating a conservative and stable operating point that limits false positives while maintaining reliable discrimination.

The relatively low standard deviations (Mean ± Std) observed overall confirm stable cross-validation behavior and reproducibility, indicating that the performance does not rely on favorable data splits. In particular, the Combined Dataset exhibits consistently lower standard deviation values compared to individual sites, reflecting strong performance consistency across folds and highlighting the robustness of the model when trained on a larger and more diverse sample. This improved stability can be attributed to the increased dataset size, which reduces sensitivity to small perturbations during cross-validation.

However, higher standard deviations are observed at certain sites (e.g., NYU precision SD = 0.138), which aligns with prior findings on the ADHD-200 dataset. Brown et al. [78] showed that NYU exhibits high variability due to substantial intra-site participant heterogeneity and class distribution shifts between training and test sets. Olivetti et al. [79] further demonstrated that inter-site batch effects, arising from differences in acquisition protocols and scanner hardware, significantly impact classifier performance across sites. Taspinar et al. [80] emphasized that intra-site heterogeneity is a key driver of unstable model performance, while [44] showed that limited samples per fold further increase cross-fold variability in ADHD classification tasks.

In contrast, site-specific datasets, characterized by smaller sample sizes, show relatively higher standard deviations, as even minor variations in fold composition may lead to noticeable fluctuations in performance. Therefore, the variability observed across sites is mainly related to dataset size and heterogeneity, whereas the Combined Dataset benefits from enhanced statistical robustness and model stability. Methodologically, the integration of PLV-based functional connectivity with GraphSAGE aggregation enables the extraction of robust node embeddings that capture local topological relationships within brain networks, thereby stabilizing connectivity representations across sites and contributing to consistent predictive performance.

These results demonstrate strong discriminative power, balanced sensitivity–specificity behavior, high AUC values, and stable generalization across multiple acquisition sites, confirming the robustness and practical reliability of the proposed framework.

Figure 4 presents the confusion matrices of the GraphSAGE classifier for each site and for the combined dataset. These matrices provide a direct view of the classification outcomes by showing the number of correctly and incorrectly predicted samples for each class.

The confusion matrices show strong classification performance, with dominant values along the main diagonal indicating that the model correctly identifies the majority of both ADHD and TDC cases across sites. NeuroIMAGE stands out with particularly low error rates (only 2 FN and 2 FP), reflecting near-perfect separation. OHSU and Peking also demonstrate robust results, with minimal FNs (2 each) suggesting high recall for ADHD detection, though Peking has a higher FP count (26), which may indicate some over-prediction of positives. For KKI, the matrix reveals balanced but moderate performance, with low misclassifications (3 FN and 2 FP) relative to the sample size, confirming reliable discrimination despite the site’s smaller dataset. NYU shows a higher volume of correct classifications (140 TP and 64 TN), but with noticeable FPs (25) and FNs (22), pointing to slight challenges in precision and recall that align with its PR and ROC patterns. The Combined Dataset matrix aggregates these outcomes effectively, with substantial correct predictions (537 TN and 263 TP) and reduced relative errors (19 FP and 76 FN), demonstrating that multisite training enhances accuracy by leveraging diverse data to minimize site-specific biases. The confusion matrices affirm GraphSAGE’s effectiveness in ADHD classification, with minimal off-diagonal errors underscoring reliable class separation. The results for individual sites highlight variability influenced by sample size and data quality, while the Combined Dataset emphasizes the advantages of integration for improved robustness and clinical applicability.

Figure 5 presents the Precision–Recall (PR) curves of the GraphSAGE model evaluated on the ADHD-200 dataset for each acquisition site and for the combined dataset. The PR representation is particularly suitable for this task because the dataset is imbalanced, and it directly reflects the trade-off between correctly identifying ADHD subjects (recall) and avoiding false positives (precision). The PR curves demonstrate strong discriminative performance across sites, with high Average Precision (AP) values. NeuroIMAGE (AP = 0.97), OHSU (AP = 0.96), Peking (AP = 0.95), and NYU (AP = 0.89) exhibit consistently high precision across a wide range of recall levels. In these datasets, precision remains close to 1.0 at lower recall values and decreases gradually as recall increases, indicating that the model effectively identifies positive cases while maintaining control over false positives.

The curves for OHSU, and Peking show a smooth and progressive decline in precision as recall approaches 1.0, suggesting stable ranking of predictions and well-calibrated probability outputs. NeuroIMAGE demonstrates particularly strong performance, with precision remaining high even at moderate-to-high recall levels, reflecting robust class separation.

In contrast, KKI (AP = 0.84) presents a less stable curve, with more pronounced fluctuations in precision as recall increases. Precision decreases more sharply at higher recall levels, indicating that when the model attempts to capture nearly all positive cases, it introduces more false positives. This behavior may be due to smaller sample sizes, increased noise, or stronger variability in acquisition protocols and subject characteristics.

For the Combined Dataset (AP = 0.93), the curve remains smooth and stable over a broad recall range. Precision stays high at moderate recall levels and decreases mainly as recall approaches its maximum. This pattern indicates strong global ranking performance and confirms that the model generalizes well when trained on a larger and more diverse population. It also suggests that combining data from multiple sites improves the robustness of the learned representations by exposing the model to more diverse connectivity patterns. As a result, the model becomes less sensitive to site-specific characteristics and achieves more consistent behavior.

The PR analysis confirms that the proposed framework maintains high precision across clinically relevant recall ranges. The consistently high AP values across most sites demonstrate reliable positive class detection, while the shape of the curves reflects stable probability estimation and robust discriminative capacity. Figure 5 indicates that GraphSAGE performs well in general, although its effectiveness remains influenced by data quality and diversity. It further highlights the importance of multisite integration for improving stability and reducing variability across datasets.

Figure 6 presents the Receiver Operating Characteristic (ROC) curves of the GraphSAGE model for each acquisition site and for the Combined Dataset. The ROC curve evaluates the trade-off between the true positive rate (sensitivity) and the false positive rate across different classification thresholds, while the Area Under the Curve (AUC) summarizes the discriminative ability of the model.

The ROC curves demonstrate strong classification performance across all sites, with high AUC values. NeuroIMAGE and OHSU achieve the highest performance (AUC = 0.97), followed by Peking (AUC = 0.96). NYU obtains an AUC value of 0.89, while KKI reaches 0.93, indicating that both exhibit comparatively lower discriminative capacity. For NeuroIMAGE and OHSU, the ROC curves rise steeply toward the top-left corner of the plot, reflecting high sensitivity even at low false positive rates. This behavior indicates excellent class separation and strong ranking of predicted probabilities. Similarly, the Peking dataset shows a smooth curve approaching the optimal region, confirming reliable discrimination between ADHD and control subjects.

In contrast, NYU and KKI present less steep initial slopes and slightly lower AUC values, suggesting that the model has more difficulty distinguishing ADHD from control subjects in these datasets. This reduced performance may be related to differences in imaging protocols, scanner properties, population characteristics, or sample size. Achieving high sensitivity in these sites requires accepting a relatively higher false positive rate. Nevertheless, the curves remain clearly above the diagonal reference line, confirming performance substantially better than random classification.

The Combined Dataset (AUC = 0.96) exhibits a smooth and stable ROC curve that closely approaches the top-left corner. This result confirms strong global discriminative performance when training and evaluating on a larger and more diverse sample. The high AUC indicates that the model maintains robust ranking ability across heterogeneous data distributions.

The ROC analysis confirms that GraphSAGE achieves high sensitivity across a broad range of false positive rates. The consistently high AUC values across sites demonstrate reliable class separation, while the Combined Dataset further highlights the benefit of multisite integration for improving generalization and stability.

#### 5.3.1. Ablation Study

This section presents an ablation study aimed at assessing the contribution of key design choices in the proposed framework. We first investigate the impact of the connectivity measure used to construct the input graphs, before examining the effect of the architectural components of the model. Correlation-based functional connectivity is one of the most widely used approaches for constructing brain networks from fMRI data. It quantifies the linear statistical dependencies between the time series of different brain regions using the Pearson correlation coefficient, and has been extensively adopted in neuroimaging research [81]. In this work, pairwise correlations between all regions of interest are computed to obtain a full correlation matrix. To derive a sparse graph from this matrix, we apply a global threshold based on the 80th percentile of the absolute correlation values, retaining only the strongest connections and discarding the weaker ones. This thresholding strategy is consistent with the one used for PLV-based graphs, ensuring a direct and fair comparison between the two connectivity approaches. Table 8 compares the performance of GraphSAGE when built on correlation-based versus PLV-based functional connectivity graphs. Both connectivity measures yield strong classification results, which confirms that GraphSAGE can effectively leverage different types of brain network representations. The correlation-based approach already performs competitively, with an accuracy of 0.874 ± 0.015 and an AUC of 0.952 ± 0.017, suggesting that Pearson correlation captures sufficient linear structure to support ADHD classification. PLV-based graphs show consistent but moderate improvements across most metrics—accuracy (0.898), precision (0.930), F1-score (0.852), and AUC (0.958)—while the gain in recall remains relatively limited (0.787 vs. 0.760). Beyond mean performance, PLV-based results tend to exhibit slightly lower standard deviations across several metrics, indicating more stable behavior across folds. These observations suggest that PLV may provide a complementary representation of functional interactions by capturing phase synchronization effects, although the overall performance differences remain moderate.

Table 9 presents an ablation study comparing adaptive threshold optimization with a fixed decision threshold of 0.5. This analysis highlights the impact of threshold selection on classification behavior and calibration in a multi-site clinical setting.

The results indicate that adaptive thresholding achieves a more balanced trade-off between precision and recall across most sites. In KKI and NYU, adaptive calibration improves accuracy and precision while maintaining competitive recall, indicating improved control of false positives without substantially reducing sensitivity. These results suggest that a fixed 0.5 threshold may not be optimal under heterogeneous data distributions.

In certain sites, such as Peking and NeuroIMAGE, the fixed threshold slightly increases recall in Peking (0.970 vs. 0.961), indicating that the optimal operating point may vary according to site-specific characteristics. Nevertheless, adaptive thresholding generally provides a more consistent balance between performance metrics, which is particularly important in clinical classification tasks where both reliability and interpretability are required.

For the Combined Dataset, recall is higher with the fixed 0.5 threshold (0.896 vs. 0.787). However, this improvement in sensitivity is associated with reduced precision and increased variability. The standard deviation values reported in Table 8 indicate that the adaptive configuration ensures more consistent performance across folds. This observation reflects a trade-off between sensitivity and precision, where the fixed threshold favors higher recall, while the adaptive strategy provides better control of false positives. In a screening-oriented context, higher recall may be preferred, whereas in settings requiring more reliable predictions, improved precision and stability can be advantageous. Therefore, the choice of threshold should be guided by the intended clinical objective rather than assuming a single optimal operating point.

These findings demonstrate that threshold selection significantly affects operating characteristics. In this context, adaptive calibration provides more stable and controlled performance across heterogeneous sites, although it does not uniformly dominate the fixed threshold across all metrics. This makes it a suitable option when prioritizing robustness and consistency in practical clinical deployment. Table 10 presents an ablation study evaluating the effect of the PLV threshold percentile on classification performance using the Combined Dataset. The results clearly show that graph sparsification plays a critical role in model effectiveness.

Among the tested configurations, the 80th percentile achieves the best performance, with the highest accuracy (0.898 ± 0.022), precision (0.930 ± 0.036), F1-score (0.852 ± 0.012), and AUC (0.958 ± 0.012). Recall is also highest at this threshold (0.787 ± 0.044), providing a balanced trade-off between sensitivity and precision.

The 60th percentile yields moderate results, while the 70th percentile leads to a noticeable decrease in accuracy, precision, and AUC. Although recall remains relatively similar across percentiles, the discriminative performance declines when the threshold is lower. This suggests that retaining a larger number of weaker functional connections may introduce noise into the graph structure, reducing the quality of learned representations.

In contrast, the 80th percentile appears to preserve the most informative connectivity patterns while removing less relevant edges. The relatively low standard deviation at this level also indicates stable behavior across folds. These findings justify the selection of the 80th percentile as the optimal configuration for graph construction. However, it is worth noting that we did not explicitly analyze site-specific PLV distributions, which may influence the behavior and generalizability of a uniform global threshold across heterogeneous acquisition sites.

Table 11 reports an ablation study analyzing the effect of GraphSAGE depth on classification performance using the Combined Dataset.

The results show a clear improvement as the number of layers increases. With only one layer, the model achieves limited performance (accuracy = 0.715 ± 0.039, AUC = 0.784 ± 0.042), indicating that shallow aggregation is insufficient to capture complex inter-regional interactions. Although recall remains moderate (0.755 ± 0.004), precision and discriminative ability remain relatively low.

Using two layers significantly improves all metrics, confirming that incorporating broader neighborhood information enhances representation learning. However, the best performance is obtained with three layers (proposed configuration), achieving the highest accuracy (0.898 ± 0.022), precision (0.930 ± 0.036), recall (0.787 ± 0.044), F1-score (0.852 ± 0.012), and AUC (0.958 ± 0.012).

Importantly, the standard deviation remains low for the three-layer model, indicating that increased depth improves performance without compromising stability. These results demonstrate that deeper message passing enables the model to better capture higher-order connectivity patterns, supporting the choice of a three-layer GraphSAGE architecture. Table 12 presents an ablation study analyzing the effect of the class weight parameter on classification performance using the Combined Dataset. The class weight is applied to the minority class in order to address the class imbalance problem and to reduce bias toward the majority class.

When no weighting is applied (class weight = 1), the model achieves moderate performance (accuracy = 0.853 ± 0.025, AUC = 0.920 ± 0.025). Although recall remains acceptable (0.772 ± 0.011), precision and F1-score are lower compared to higher weighting configurations, indicating that the model does not sufficiently emphasize the minority ADHD class.

Increasing the class weight to 1.5 improves performance, particularly in terms of precision (0.923 ± 0.069) and AUC (0.954 ± 0.028). This suggests that assigning greater importance to the minority class helps the model better distinguish ADHD subjects. However, the variability across folds slightly increases in this configuration.

With a class weight of 2, recall slightly decreases (0.755 ± 0.003), and the performance does not surpass that of the 1.5 configuration, indicating that moderate reweighting alone does not guarantee optimal balance.

The best results are obtained with a class weight of 3 (proposed configuration), which achieves the highest accuracy (0.898 ± 0.022), precision (0.930 ± 0.036), recall (0.787 ± 0.044), F1-score (0.852 ± 0.012), and AUC (0.958 ± 0.012). Importantly, this configuration also shows low standard deviation, reflecting stable and consistent behavior across folds.

These findings confirm that applying an appropriate class weight to the minority class effectively mitigates the impact of class imbalance. A higher class weight provides the most balanced and stable performance, demonstrating that emphasizing ADHD samples during training improves discriminative ability without compromising generalization.

#### 5.3.2. Comparison with Other Methods

Table 13 presents a comparative analysis of classification accuracy across ADHD-200 acquisition sites. The comparison includes several previously published methods and the proposed PLV-GraphSAGE approach.

Early approaches such as FCNet and DeepFMRI report moderate average accuracies (60.4% and 67.9%, respectively), indicating limited generalization across sites. The 3D-CNN method improves performance, achieving an average accuracy of 71.6%, but still shows variability between datasets.

More recent approaches demonstrate stronger performance. Dual Subspace Learning achieves high accuracy in NYU (92.4%) and Peking (89.4%), with an average of 87.1%. The attention attribute-enhanced network also reports strong results, particularly in KKI (94.5%) and NeuroIMAGE (98.4%), reaching an average accuracy of 86.2%.

The proposed PLV-GraphSAGE model achieves the highest average accuracy (89.9%) across sites. It delivers competitive or superior performance in KKI (94.7%) and OHSU (93.9%), and maintains strong results in NeuroIMAGE (92.0%) and Peking (87.7%). Although NYU accuracy (81.6%) does not exceed the best reported value, it remains competitive and consistent with the overall trend.

Importantly, PLV-GraphSAGE demonstrates stable performance across multiple sites without extreme fluctuations. Unlike some previous methods that achieve very high performance in specific datasets but show inconsistency across others, the proposed model maintains balanced accuracy across heterogeneous acquisition conditions.

The results confirm that integrating PLV-based connectivity with GraphSAGE representation learning provides strong generalization across sites. The higher average accuracy highlights the robustness of the proposed framework and supports its effectiveness for multi-site ADHD classification. Table 14 represents the comparative performance of various methods on the ADHD-200 dataset, evaluated using common metrics including Accuracy, Recall, and Specificity. The results reveal considerable variability across approaches.

Conventional and early deep learning approaches show moderate performance. BrainNetCNN [83] achieves 63.77% accuracy, while MDCN [85] reports 67.45% reflecting limitations in capturing complex brain connectivity patterns with conventional architectures. The LSTM with spatio-temporal convolution model reaches 71.3% accuracy, reflecting improvements brought by temporal modeling. The CNN approach proposed by De Silva et al. achieves 85.36% accuracy but with relatively lower recall (72.8%) and specificity (66.54%), indicating class imbalance in prediction. TLNN [84] reports strong recall (90.0%), though specificity remains lower (77.0%). CAMEL [45] attains 86.7% accuracy; however, missing recall and specificity values limit detailed comparison. USMDA [43] achieves 84.38% accuracy and 83.87% recall, highlighting the effectiveness of unsupervised multisource domain adaptation for ADHD classification.

Graph-based approaches, including GCN [47] and BrainGNN [48], delivered intermediate performance, showing the advantages of modeling the brain as a graph but also indicating that further enhancements are required to fully leverage graph structures. Recent hybrid models, such as HAGCN [52], achieved competitive results, with an accuracy of 77.95% and recall of 80.98%, suggesting that multi-head attention mechanisms can improve classification performance.

The proposed PLV-GraphSAGE method outperforms all previously reported approaches, achieving the highest accuracy (89.9%) while maintaining comparable recall (78.7%) and specificity (96.5%). This demonstrates that integrating phase-locking value (PLV) connectivity features with GraphSAGE provides a more discriminative representation of functional brain networks. Nonetheless, there is still potential for improvement, particularly in enhancing recall further without compromising specificity. It is also worth noting that some studies report missing values (NA) for certain metrics, limiting direct comparison for those specific measures. However, this comparison should be interpreted with caution, as differences in preprocessing steps, feature extraction methods, and evaluation protocols can significantly influence the reported results and make direct comparisons challenging. In general, modern graph-based and hybrid approaches, particularly PLV-GraphSAGE, show strong effectiveness for ADHD classification.

### 5.4. Limitations and Future Work

The PLV-GraphSAGE model demonstrates strong performance for ADHD classification on the ADHD-200 dataset, achieving high accuracy (89.2%) and AUC (96.4%) on the combined dataset. However, several limitations should be considered. The model exhibits site-dependent variability, reflecting differences in imaging protocols, scanner characteristics, demographic distributions, and sample sizes across centers. In the combined dataset, the recall remains moderate, indicating that further improvement is needed to enhance sensitivity, particularly for the positive (ADHD) class. Furthermore, the current framework relies exclusively on functional connectivity representations derived from PLV graphs and does not yet incorporate structural or multimodal information. The model has also not been evaluated on external datasets, which limits conclusions about its generalization beyond ADHD-200. In addition, the current evaluation protocol is based on pooled cross-validation, where data from different sites are mixed across training and test folds. This setting does not fully reflect true out-of-site generalization, since the model is not evaluated on completely unseen sites.

Future work will focus on addressing these aspects by incorporating inter-site harmonization strategies to reduce variability; extending the framework to integrate multimodal data, including structural MRI, EEG, or clinical features; and exploring advanced architectures with attention mechanisms or ensemble strategies to improve recall model performance. In addition, federated learning approaches will be investigated to enable training across distributed datasets while preserving data privacy. External validation on independent datasets, combined with data augmentation strategies, will be considered to further assess generalization. Finally, efforts will be made to optimize computational efficiency to facilitate practical and scalable clinical deployment.

## 6. Conclusions

This study introduces a graph-based deep learning framework for automated ADHD classification using resting-state fMRI data. By combining phase-locking value (PLV) functional connectivity with the GraphSAGE representation learning model, the proposed method effectively captures regional brain activity and inter-regional interactions within a unified framework. This integration enables the model to learn discriminative, connectivity-aware features while reducing the impact of signal variability and inter-subject differences.

The PLV-GraphSAGE framework demonstrates strong performance for ADHD classification on the ADHD-200 dataset. Experimental evaluations were conducted across five acquisition sites as well as on a combined multi-site dataset. The results show consistent performance across individual sites, suggesting the model’s ability to handle variability across heterogeneous imaging conditions. These findings are further supported by the average results across sites, which remain competitive with existing approaches. On the combined dataset, the model achieved 89.2% accuracy, 96.4% AUC, and 96.0% specificity, indicating stable and discriminative performance in multi-site settings. In addition, a comparative analysis with Pearson correlation-based connectivity shows that PLV yields slightly improved performance, suggesting the benefit of phase-based functional connectivity for capturing informative brain interactions.

The proposed PLV-GraphSAGE framework provides an efficient and scalable approach for automated ADHD classification from rs-fMRI data, and represents a promising step toward early detection and data-driven analysis of neurodevelopmental disorders.

## Figures and Tables

**Table 1:** Summary of Machine Learning Approaches for ADHD Classification.

Study	Year	Data Type	Main Features	ML Method	Accuracy
Sen et al. [15]	2018	Structural MRI + rs-fMRI	Structural textures + functional connectivity	Linear SVM	67.3%
Chen et al. [16]	2020	rs-fMRI	Individual functional connectivity patterns	SVM (two-step)	89.4%
Rostami et al. [17]	2020	Behavioral + neuropsychological + EEG	Sensory-motor + cognitive features	Decision Tree	Control vs. ADHD: 100%; Subtype classification: 80.41%, 84.17%, 71.46%
Lohani et al. [18]	2023	Structural MRI	Gray matter volume, cortical thickness	SVM	75%
Lin et al. [19]	2023	Multimodal MRI (ABCD)	DTI + structural MRI + rs-fMRI	Multiple Kernel Learning	AUC = 0.613
Zamanzadeh et al. [20]	2024	rs-fMRI (ADHD-200)	Graph-theoretical features	EEC/XGBoost/BRF	EEC: 74.3%, XGBoost: 72.7%, BRF: 68.0%
Alsharif et al. [21]	2024	Event-Related Potentials (ERP)	Electrophysiological features	SVM	91%

**Table 2:** Summary of Deep Learning Approaches for ADHD Classification Using fMRI.

Study	Year	Data Type	Main Features	DL Method	Accuracy
Zou et al. [12]	2017	fMRI + sMRI	Spatial patterns from structural and functional MRI	3D-CNN	70.50%
Mao et al. [24]	2019	fMRI	Spatiotemporal features	Conv + RNN	71.3%
Zhang et al. [27]	2020	rs-fMRI	Multi-site datasets	Separated Channel Attention CNN	68.6%
Riaz et al. [2]	2020	rs-fMRI	Raw fMRI sequences	DeepFMRI (CNN + RNN)	73.1%
Gao et al. [29]	2020	rs-fMRI	Attention attribute-enhanced network	VAE + Attention	NYU: 76.42%, Peking: 78.43%, KKI: 94.54%, NeuroIMAGE: 98.40%
Qiang et al. [33]	2021	fMRI	Functional brain network mapping	Deep VAE	71.3%
De Silva et al. [35]	2021	rs-fMRI	Seed-based, fALFF, ReHo features	CNN	85.36–86%
Qiang et al. [38]	2022	rs-fMRI	Spatiotemporal attention autoencoders	Attention Autoencoder	72.5%
Qin et al. [41]	2022	rs-fMRI	Spatio-temporal + phenotypic features	Trans3D-Ensemble (3D-CNN + Transformer + RF)	74.5%
Hsieh et al. [42]	2023	rs-fMRI	Seed-correlation features	Data-driven method	83.24%
Chen et al. [3]	2023	rs-fMRI	Biomarker detection with attention autoencoder	Attention Autoencoder	73.2%
Mengi et al. [43]	2024	sMRI + fMRI	Multi-source domain adaptation	Unsupervised DL	84.38%
Xue et al. [45]	2025	rs-fMRI	Topological manifold learning	Manifold DL	86.7%

**Table 3:** Summary of Graph-Based Approaches for ADHD Classification Using rs-fMRI.

Study	Year	Data Type	Main Features	ML Method	Accuracy
Parisot et al. [47]	2018	rs-fMRI/MRI	Population graph, inter-subject phenotypic similarities	GCN	61.02%
Li et al. [48]	2021	rs-fMRI	Individual brain graphs, node/edge selection for interpretability	BrainGNN	66.63%
Zhao et al. [49]	2022	rs-fMRI	Dynamic functional connectivity, time-varying brain networks	Dynamic GCN	72.0%
Zhang et al. [50]	2023	rs-fMRI	Dynamic graph library, contrastive graph representations	Adversarial Graph Contrastive Learning (A-GCL)	70.92%
Hu et al. [51]	2024	rs-fMRI	Functional connectivity between brain regions	GCN	84.49%
Wu et al. [52]	2025	rs-fMRI	Hybrid-order brain network topology (local and higher-order features)	HAGCN (GCN + attention)	77.95%

**Table 4:** Distribution of participants across acquisition sites.

Site	Total	ADHD	TDC
Kennedy Krieger Institute (KKI)	94	25	69
New York University (NYU)	251	146	105
NeuroIMAGE (NI)	48	25	23
Oregon Health & Science University (OHSU)	113	43	70
Peking University (PU)	245	102	143
Full Data (KKI, NYU, NI, OHSU, PU, Pittsburgh, WashU)	895	341	554

**Table 5:** Overview of Extracted Node-Level Features.

Feature	Description
Mean, Std. Dev.	Central tendency and dispersion
Skewness, Kurtosis	Distribution shape (non-Gaussianity)
RMS, Energy	Signal amplitude and power
Zero crossings	Temporal complexity/oscillatory rate
Lag-1 autocorrelation	Short-term temporal dependency
Spectral entropy	Frequency-domain irregularity
Mean frequency	Power-weighted spectral centroid
Wavelet energy (db4, L3)	High-frequency time–frequency energy
25th, 75th percentiles	Robust amplitude range

**Table 6:** Summary of Training Settings.

Parameter	Value
Cross-validation	5-fold
Epochs	100/200
Early stopping patience	15/30
Optimiser	Adam
Learning rate	$1\times 10^{-3}$
Weight decay	$1\times 10^{-5}$/$1\times 10^{-3}$
Loss function	Weighted cross-entropy
Scheduler	ReduceLROnPlateau
Scheduler factor	0.5
Scheduler patience	10
Class weight multiplier	3.0
Minimum recall target	0.75
Gradient clipping	Max-norm = 1.0
Batch size	16

**Table 7:** Classification performance of the proposed PLV-GraphSAGE model across sites (Mean ± Std).

Site	Accuracy	Precision	Recall	Specificity	F1-Score	AUC
KKI	0.947 ± 0.033	0.927 ± 0.090	0.880 ± 0.098	0.971 ± 0.090	0.897 ± 0.064	0.900 ± 0.099
NYU	0.816 ± 0.121	0.849 ± 0.138	0.862 ± 0.062	0.719 ± 0.012	0.850 ± 0.090	0.818 ± 0.094
OHSU	0.939 ± 0.081	0.913 ± 0.129	0.953 ± 0.058	0.928 ± 0.090	0.929 ± 0.088	0.944 ± 0.092
Peking	0.877 ± 0.116	0.878 ± 0.107	0.961 ± 0.047	0.818 ± 0.076	0.897 ± 0.088	0.905 ± 0.092
NeuroIMAGE	0.920 ± 0.098	0.933 ± 0.123	0.920 ± 0.088	0.913 ± 0.062	0.923 ± 0.067	0.920 ± 0.107
Combined Dataset	0.898 ± 0.022	0.930 ± 0.036	0.787 ± 0.044	0.965 ± 0.032	0.852 ± 0.012	0.958 ± 0.012
**Average**	**0.900**	**0.905**	**0.894**	**0.886**	**0.891**	**0.908**

**Table 8:** GraphSAGE Performance Using PLV- and Correlation-Based Functional Connectivity for ADHD Classification.

Connectivity Approach	Accuracy	Precision	Recall	F1-Score	AUC
Correlation-Based	0.874 ± **0.015**	0.896 ± 0.0417	0.7605 ± 0.056	0.8224 ± 0.0182	0.9520 ± 0.0170
PLV-Based	**0.898** ± 0.022	**0.930** ± **0.036**	**0.787** ± **0.044**	**0.846** ± **0.012**	**0.958** ± **0.012**

**Table 9:** Comparison between Adaptive and Fixed (0.5) Thresholds (Mean ± Std).

Dataset	Threshold	Accuracy	Precision	Recall	F1-Score	AUC
KKI	Adaptive	**0.947** ± 0.033	**0.927** ± 0.090	0.880 ± 0.098	**0.897** ± 0.064	0.900 ± 0.099
	Fixed 0.5	0.926 ± **0.025**	0.820 ± **0.016**	**0.920** ± 0.098	0.865 ± **0.053**	
NYU	Adaptive	**0.816** ± **0.121**	**0.849** ± 0.138	**0.862** ± **0.062**	**0.850** ± **0.090**	0.818 ± 0.094
	Fixed 0.5	0.764 ± 0.162	0.849 ± **0.114**	0.704 ± 0.094	0.760 ± 0.117	
OHSU	Adaptive	**0.939** ± **0.081**	**0.913** ± **0.129**	0.953 ± **0.058**	**0.929** ± **0.088**	0.944 ± 0.092
	Fixed 0.5	0.912 ± 0.095	0.851 ± 0.137	**0.956** ± 0.089	0.898 ± 0.110	
Peking	Adaptive	**0.877** ± 0.116	0.878 ± 0.107	0.961 ± 0.047	**0.897** ± 0.088	0.905 ± 0.092
	Fixed 0.5	0.857 ± **0.044**	0.819 ± **0.076**	**0.970** ± **0.024**	**0.874** ± **0.041**	
NeuroIMAGE	Adaptive	**0.920** ± 0.098	0.933 ± 0.123	**0.920** ± **0.088**	**0.923** ± **0.067**	0.920 ± 0.107
	Fixed 0.5	0.895 ± **0.068**	0.902 ± **0.076**	0.920 ± 0.098	0.904 ± 0.083	
Combined	Adaptive	**0.898** ± **0.022**	**0.930** ± **0.036**	0.787 ± **0.044**	0.852 ± **0.012**	0.958 ± 0.012
	Fixed 0.5	0.891 ± 0.040	0.838 ± 0.072	**0.896** ± 0.046	**0.864** ± 0.033	

**Table 10:** Comparison of Classification Metrics by PLV Threshold Percentile (Mean ± Std) on Combined Dataset.

PLV Percentile	Accuracy	Precision	Recall	F1-Score	AUC
60th	0.870 ± 0.032	0.888 ± 0.079	0.762 ± 0.009	0.819 ± 0.037	0.945 ± 0.028
70th	0.822 ± 0.045	0.780 ± 0.094	0.763 ± **0.006**	0.769 ± 0.045	0.894 ± 0.041
80th	**0.898** ± **0.022**	**0.930** ± **0.036**	**0.787** ± 0.044	**0.846** ± **0.012**	**0.958** ± **0.012**

**Table 11:** Ablation Study on GraphSAGE Depth (Combined Dataset, Mean ± Std).

GraphSAGE Layers	Accuracy	Precision	Recall	F1-Score	AUC
1 Layer	0.715 ± 0.039	0.605 ± 0.051	0.755 ± 0.004	0.671 ± 0.031	0.784 ± 0.042
2 Layers	0.800 ± 0.022	0.733 ± 0.039	0.754 ± **0.006**	0.743 ± 0.022	0.873 ± 0.025
3 Layers (Proposed)	**0.898** ± 0.022	**0.930** ± **0.036**	**0.787** ± 0.044	**0.846** ± **0.012**	**0.958** ± **0.012**

**Table 12:** Ablation Study on Class Weight Parameter (Combined Dataset, Mean ± Std).

Class Weight	Accuracy	Precision	Recall	F1-Score	AUC
1	0.853 ± 0.025	0.834 ± 0.053	0.772 ± 0.011	0.801 ± 0.029	0.920 ± 0.025
1.5	0.889 ± 0.031	0.923 ± 0.069	0.780 ± 0.034	0.844 ± 0.039	0.954 ± 0.028
2	0.853 ± 0.038	0.852 ± 0.089	0.755 ± **0.003**	0.799 ± 0.041	0.931 ± 0.034
3	**0.898** ± **0.022**	**0.930** ± **0.036**	**0.787** ± 0.044	**0.846** ± **0.012**	**0.958** ± **0.012**

**Table 13:** Comparative analysis of classification performance across ADHD-200 sites using accuracy.

Author	Method	NYU	Peking	KKI	NI	OHSU	Average
Riaz et al. [82], 2017	FCNet	58.5%	62.7%	–	60.0%	–	60.4%
Zou et al. [12], 2017	3D-CNN	70.5%	–	72.8%	–	–	71.6%
Riaz et al. [2], 2020	DeepFMRI	73.1%	62.7%	–	67.9%	–	67.9%
Chen et al. [16], 2020	Dual Subspace Learning	**92.4%**	**89.4%**	85.5%	81.2%	–	87.1%
Gao et al. [29], 2020	Attention attribute-enhanced network	76.4%	78.4%	94.5%	**98.4%**	83.3%	86.2%
**Ours**	PLV-GraphSAGE	81.6%	87.7%	**94.7%**	92.0%	**93.9%**	**89.9%**

**Table 14:** Comparative analysis on ADHD-200 combined dataset using common evaluation metrics.

Author	Method	Accuracy (%)	Recall (%)	Specificity (%)
Kawahara et al. [83], 2017	BrainNetCNN	63.77	69.87	58.37
Parisot et al. [47], 2018	GCN	61.02	46.48	70.12
Mao et al. [24], 2019	LSTM + spatio-temporal conv.	71.3	73.2	69.7
De Silva et al. [35], 2021	CNN	85.36	72.8	66.54
Li et al. [48], 2021	BrainGNN	66.63	70.10	63.97
Meng et al. [84], 2022	TLNN	82.0	**90.0**	77.0
Yang et al. [85], 2023	MDCN	67.45	71.97	62.39
Mengi et al. [43], 2024	USMDA	84.38	83.87	NA
Zamanzadeh et al. [20], 2024	EEC	74.30	64.28	78.43
Wu et al. [52], 2025	DNN	62.91	66.51	58.61
Xue et al. [45], 2025	CAMEL	86.7	NA	NA
Wu et al. [52], 2025	HAGCN	77.95	80.98	74.25
**Ours**	PLV-GraphSAGE	**89.8**	78.7	**96.5**

NA: Not Available.

## Data Availability

The data used in this study are publicly available from the ADHD-200 Preprocessed initiative (Athena pipeline): http://preprocessed-connectomes-project.org/adhd200/ (accessed on 15 February 2025), The code is publicly available at: https://github.com/Rabab070707/ADHD_PLV_GraphSAGE/ (accessed on 13 May 2026).

## Supplementary Materials

The following supporting information can be downloaded at: https://www.mdpi.com/article/10.3390/bioengineering13050586/s1, File S1: Source code for the graph-based ADHD classification using GraphSAGE.

## Abbreviations

The following abbreviations are used in this manuscript:

ADHD	Attention Deficit Hyperactivity Disorder
fMRI	Functional Magnetic Resonance Imaging
rs-fMRI	Resting-State Functional Magnetic Resonance Imaging
PLV	Phase-Locking Value
EEG	Electroencephalography
CNN	Convolutional Neural Network
GCN	Graph Convolutional Network
GraphSAGE	Graph Sample and Aggregate
