# Supplementary material for: Robust Multi-Site ADHD Classification via GraphSAGE-Based Functional Connectivity Modeling from rs-fMRI
Source: Bioengineering (Basel). 2026 May 20;13(5):586. doi: 10.3390/bioengineering13050586 (PMC13203753; doi:10.3390/bioengineering13050586)
Supplement: Supplementary file 1 [file bioengineering-13-00586-s001.zip › bioengineering-4271001-supplementary.pdf]

Article

Robust Multi-Site ADHD Classification via GraphSAGE-Based Functional Connectivity Modeling from rs-fMRI

Rabab BOUSMAHA [1]*, Khouloud MERIBAI [1], Nardjes BOUCHEMAL [2,3], Naila BOUCHEMAL [4], and GALINA IVANOVA [5]

[1] LabRi Laboratory, Ecole Superieure en Informatique, Sidi Bel Abbes 22000, Algeria; r.bousmaha@esi-sba.dz , k.meribai@esi-sba.dz
[2] LIRE Laboratory, Abdelhamid Mehri Constantine 2 University, Constantine 25000, Algeria; n.bouchemal.dz@ieee.org
[3] LISI Laboratory of Intelligent Systems and Informatics, Mila 43000, Algeria; n.bouchemal.dz@ieee.org
[4] LyRIDS ECE Ecole d'Ingénieurs de Paris, 75015 Paris, France; naila.bouchemal@ece.fr
[5] Faculty of Electrical Engineering, Electronics and Automation, University of Ruse "Angel Kanchev", 7017 Ruse, Bulgaria; giivanova@uni-ruse.bg
* Correspondence: r.bousmaha@esi-sba.dz.

1. PROPOSED METHOD

1.1. Data Augmentation

To improve model generalization, two augmentation strategies were applied: additive Gaussian noise and temporal shifting.

1.1.1. Additive Gaussian Noise

Gaussian noise is added to the original time series to simulate measurement variability:

Listing 1: Additive Gaussian Noise

```
def augment_add_noise(ts_df, sigma=0.01):
    return ts_df + np.random.normal(0, sigma, ts_df.shape)
```

1.1.2. Time Shift Augmentation

Temporal shifting is applied using a circular shift to preserve statistical properties while modifying temporal alignment:

Listing 2: Time Shift Augmentation

```
def augment_time_shift(ts_df, shift=5):
    shifted = np.roll(ts_df.values, shift=shift, axis=0)
    return pd.DataFrame(shifted, columns=ts_df.columns)
```

1.2. Feature Extraction

In this section, we describe the extracted features used in the proposed model. A total of 13 features are computed from the ROI time series of resting-state fMRI (rs-fMRI), capturing statistical, spectral, and dynamical characteristics of the BOLD signal.

Listing 3: Feature Extraction

```
import numpy as np
def extract_node_features(signal, fs=0.5):
    feats = []
```

```
    signal = np.asarray(signal).ravel()

    # Mean
    feats.append(np.mean(signal))

    # Standard Deviation
    feats.append(np.std(signal))

    # Skewness
    feats.append(skew(signal))

    # Kurtosis
    feats.append(kurtosis(signal))

    # Root Mean Square (RMS)
    feats.append(np.sqrt(np.mean(signal**2)))

    # Energy
    feats.append(np.sum(signal**2))

    # Zero crossings
    feats.append(int(np.sum(np.abs(np.diff(np.sign(signal))) > 0)))

    # --- Frequency-domain features ---
    freqs, psd = welch(signal, fs=fs, nperseg=min(64, len(signal)))
    psd_sum = np.sum(psd)
    psd_norm = psd / psd_sum if psd_sum > 0 else psd

    # Spectral entropy (frequency-domain irregularity)
    feats.append(entropy(psd_norm) if psd_sum > 0 else 0)

    # Mean frequency (power-weighted spectral centroid)
    feats.append(np.sum(freqs * psd_norm) / np.sum(psd_norm) if
        psd_sum > 0 else 0)

    # --- Time-frequency feature ---
    try:
        coeffs = pywt.wavedec(signal, 'db4', level=3)

        # Wavelet energy (db4, level 3)
        feats.append(np.sum(np.square(coeffs[-1])) if len(coeffs) > 0
            else 0)
    except Exception:
        feats.append(0)

    # Lag-1 autocorrelation (short-term dependency)
    if len(signal) > 1:
        feats.append(np.corrcoef(signal[:-1], signal[1:])[0, 1])
    else:
        feats.append(0)

    # --- Robust amplitude range ---
    # 25th percentile
    feats.append(np.percentile(signal, 25))

```

```
    # 75th percentile
    feats.append(np.percentile(signal, 75))

    return np.array(feats, dtype=float)
```

1.3. Functional Connectivity and Graph Construction

1.3.1. Phase Extraction

The instantaneous phase of each ROI signal is computed using the Hilbert transform:

```
def compute_phase(signal):
    analytic = hilbert(signal)
    return np.angle(analytic)
```

1.3.2. Phase Locking Value (PLV)

PLV measures phase synchronization between two signals:

```
def compute_plv(sig1, sig2):
    ph1 = compute_phase(sig1)
    ph2 = compute_phase(sig2)
    d = ph1 - ph2
    return float(np.abs(np.mean(np.exp(1j * d))))
```

1.3.3. PLV Matrix Construction

Pairwise PLV values are computed to form a symmetric connectivity matrix:

```
def generate_plv_matrix(df):
    n = df.shape[1]
    mat = np.zeros((n, n), dtype=np.float32)
    for i in range(n):
        for j in range(i, n):
            val = compute_plv(df.iloc[:, i].values, df.iloc[:, j].
                values)
            mat[i, j] = val
            mat[j, i] = val
    return mat
```

1.4. Graph Construction

1.4.1. Adjacency Matrix Construction

Functional brain graphs were constructed from PLV matrices using a global thresholding strategy.

```
def plv_to_adj(plv_mat, threshold=None, global_threshold=None):
    n = plv_mat.shape[0]

    # Select threshold
    thr = global_threshold if global_threshold is not None else
        threshold

    # Binary adjacency matrix
    adj = (np.abs(plv_mat) >= thr).astype(int)

    # Remove self-connections
    np.fill_diagonal(adj, 0)
```

```python

    return adj
```

1.4.2. GraphSAGE Data Preparation

Graph data were converted into PyTorch Geometric format for training:

```python
def build_gcn_data_list(subject_ids, node_features_dict,
                        adj_dict, extra_graph_features=None,
                        scaler=None):

    data_list = []

    for sid in subject_ids:

        # Node-level features (ROI features)
        node_feats = node_features_dict[sid]

        # Optional graph-level features
        if extra_graph_features is not None:
            extra = extra_graph_features[sid]
            x = np.concatenate([node_feats, extra], axis=1)
        else:
            x = node_feats

        # Feature normalization
        if scaler is not None:
            x = scaler.transform(x)

        x = torch.tensor(x, dtype=torch.float)

        # Convert adjacency matrix to edge index format
        adj = adj_dict[sid]
        rows, cols = np.where(adj > 0)

        # Handle empty graphs
        if rows.size == 0:
            rows = np.arange(x.shape[0])
            cols = np.arange(x.shape[0])

        edge_index = torch.tensor(
            np.vstack([
                np.concatenate([rows, cols]),
                np.concatenate([cols, rows])
            ]),
            dtype=torch.long
        )

        # Graph label
        y = augmented_labels[sid]

        data_list.append(
            Data(
                x=x,
                edge_index=edge_index,
                y=torch.tensor([y], dtype=torch.long)
```

```
            )
        )

    return data_list
```

1.4.3. Proposed Model

The proposed GraphSAGE-based model consists of three GraphSAGE convolutional layers followed by a fully connected classifier, with Layer Normalization for improved training stability.

```
class ProposedGraphSAGE(nn.Module):

    def __init__(self, in_feats, hidden=64, num_classes=2, dropout
        =0.3):
        super().__init__()

        self.conv1 = SAGEConv(in_feats, hidden)
        self.ln1 = nn.LayerNorm(hidden)

        self.conv2 = SAGEConv(hidden, hidden)
        self.ln2 = nn.LayerNorm(hidden)
        self.conv3 = SAGEConv(hidden, hidden)
        self.ln3 = nn.LayerNorm(hidden)
        self.classifier = nn.Sequential(
            nn.Linear(hidden, hidden // 2),
            nn.ReLU(),
            nn.Dropout(dropout),
            nn.Linear(hidden // 2, num_classes)
        )

    def forward(self, data):
        x, edge_index, batch = data.x, data.edge_index, data.batch

        x = self.conv1(x, edge_index)
        x = self.ln1(x)
        x = F.relu(x)
        x = F.dropout(x, p=0.3, training=self.training)

        x = self.conv2(x, edge_index)
        x = self.ln2(x)
        x = F.relu(x)
        x = F.dropout(x, p=0.3, training=self.training)

        x = self.conv3(x, edge_index)
        x = self.ln3(x)
        x = F.relu(x)

        x = global_mean_pool(x, batch)
        return self.classifier(x)
```

1.4.4. Adaptive Threshold Optimisation

The classification threshold is selected via grid search over $[0.01, 0.99]$ rather than fixed at the conventional value of 0.5, ensuring that recall does not fall below $r_{\text{min}} = 0.75$:

```python

def select_best_threshold_variant(y_true, y_prob, min_recall=0.8):
    best_th, best_score = 0.5, -np.inf
    ths = np.linspace(0.01, 0.99, 99)
    for th in ths:
        preds = (np.array(y_prob) >= th).astype(int)
        prec = precision_score(y_true, preds, zero_division=0)
        rec = recall_score(y_true, preds, zero_division=0)
        f1v = f1_score(y_true, preds, zero_division=0)
        score = prec if rec >= min_recall else -np.inf
        if score > best_score:
            best_score = score
            best_th = th
    return best_th, best_score
```

1.4.5. Training and Evaluation Loop

The training loop handles data scaling, class-weight computation, model optimisation, early stopping, and threshold selection within each cross-validation fold:

```python
def train_and_evaluate_gcn(
    train_node_features_dict, train_adj_dict, train_labels_dict,
    val_node_features_dict, val_adj_dict, val_labels_dict,
    n_epochs=200, batch_size=16, lr=1e-3, class_weight_multiplier=3.0,
    threshold_mode='precision_at_min_recall', min_recall_target=0.75
):
    device = torch.device('cuda' if torch.cuda.is_available() else 'cpu')

    # --- Prepare data loaders ---
    train_sids = list(train_node_features_dict.keys())
    val_sids   = list(val_node_features_dict.keys())
    all_x_train = np.vstack([train_node_features_dict[sid] for sid in train_sids])
    scaler = StandardScaler().fit(all_x_train)

    train_data_list = build_gcn_data_list(train_sids, train_node_features_dict,
                                          train_adj_dict, train_labels_dict, scaler=scaler)
    val_data_list   = build_gcn_data_list(val_sids, val_node_features_dict,
                                          val_adj_dict, val_labels_dict, scaler=scaler)

    train_loader = DataLoader(train_data_list, batch_size=batch_size, shuffle=True)
    val_loader   = DataLoader(val_data_list,   batch_size=batch_size, shuffle=False)

    # --- Dynamic class-weight computation --- Equation 20
```

```
    train_labels   = np.array([train_labels_dict[sid] for sid in train_sids])
    unique_classes = np.unique(train_labels)
    cw = compute_class_weight('balanced', classes=unique_classes, y=train_labels)
    cw = list(cw)
    if len(cw) == 2:
        counts       = [(train_labels == c).sum() for c in unique_classes]
        minority_idx = np.argmin(counts)
        cw[minority_idx] *= class_weight_multiplier

    class_weights = torch.tensor(cw, dtype=torch.float).to(device)

    # --- Initialise model, optimiser, scheduler, and loss criterion ---
    in_feats   = train_data_list[0].x.shape[1]
    model      = ProposedGraphSAGE(in_feats=in_feats, hidden=64, num_classes=2).to(device)
    optimizer = Adam(model.parameters(), lr=lr, weight_decay=1e-5)
    scheduler = ReduceLROnPlateau(optimizer, mode='min', factor=0.5, patience=10)
    criterion = nn.CrossEntropyLoss(weight=class_weights)

    # --- Training loop with early stopping ---
    best_val_loss     = float('inf')
    best_model_state = None
    histories         = {'train_loss': [], 'val_loss': [], 'val_acc': []}
    patience_counter = 0
    patience          = 30
    min_delta         = 0.001  # minimum improvement to reset patience

    for epoch in range(n_epochs):
        model.train()
        train_loss = 0
        for batch in train_loader:
            batch = batch.to(device)
            optimizer.zero_grad()
            out  = model(batch)
            loss = criterion(out, batch.y)
            loss.backward()
            optimizer.step()
            train_loss += loss.item()
        train_loss /= len(train_loader)
        histories['train_loss'].append(train_loss)

        # Validation pass
        model.eval()
        val_loss = 0
        y_true, y_prob = [], []
        with torch.no_grad():
            for batch in val_loader:
                batch = batch.to(device)
                out   = model(batch)
```

```
                loss  = criterion(out, batch.y)
                val_loss += loss.item()
                probs = F.softmax(out, dim=1)[:, 1].cpu().numpy()
                y_prob.extend(probs)
                y_true.extend(batch.y.cpu().numpy())
        val_loss /= len(val_loader)
        histories['val_loss'].append(val_loss)
        val_acc = accuracy_score(y_true, (np.array(y_prob) >= 0.5).astype(int))
        histories['val_acc'].append(val_acc)

        scheduler.step(val_loss)

        if epoch % 10 == 0 or epoch == n_epochs - 1:
            print(f"Epoch {epoch+1:3d}: Train={train_loss:.4f}, "
                  f"Val={val_loss:.4f}, Acc={val_acc:.4f}")

        # Early stopping check
        if val_loss < best_val_loss - min_delta:
            best_val_loss    = val_loss
            best_model_state = model.state_dict()
            best_epoch_info  = {'epoch': epoch, 'val_loss': val_loss}
            patience_counter = 0
        else:
            patience_counter += 1
            if patience_counter >= patience:
                print(f"Early stopping triggered at epoch {epoch+1}")
                break

    # --- Load best checkpoint and run final evaluation ---
    model.load_state_dict(best_model_state)
    model.eval()
    y_true, y_prob = [], []
    with torch.no_grad():
        for batch in val_loader:
            batch = batch.to(device)
            out   = model(batch)
            probs = F.softmax(out, dim=1)[:, 1].cpu().numpy()
            y_prob.extend(probs)
            y_true.extend(batch.y.cpu().numpy())

    # Apply adaptive threshold optimisation
    best_th, _ = select_best_threshold_variant(y_true, y_prob,
                                               min_recall=min_recall_target)
    y_pred = (np.array(y_prob) >= best_th).astype(int)

    # Compute final metrics
    metrics = {
        'accuracy':  accuracy_score(y_true, y_pred),
        'precision': precision_score(y_true, y_pred, zero_division=0),
        'recall':    recall_score(y_true, y_pred, zero_division=0),
        'f1':        f1_score(y_true, y_pred, zero_division=0),
        'roc_auc':   roc_auc_score(y_true, y_prob)
```

```
    }

    return metrics, model, scaler, best_epoch_info, y_true, y_pred,
        y_prob, histories
```

1.5. Cross-Validation Pipeline (MAIN)

The main pipeline implements a 5-fold cross-validation strategy where, at each fold, 4 folds are used for training and validation and the remaining fold is held out for testing. The threshold learned on the validation set is transferred to the held-out test set:

1.6. Cross-Validation Pipeline

The main pipeline implements a nested 5-fold cross-validation strategy, where the threshold learned on the validation set is transferred to the held-out test set:

```
# --- Hyperparameters ---
N_SPLITS, N_EPOCHS, BATCH_SIZE = 5, 200, 16
LR, CLASS_WEIGHT_MULTIPLIER, PLV_PERCENTILE = 1e-3, 3, 80

# [data loading and label extraction ...]


subject_ids = list(ts_data.keys())
y = np.array([labels_dict[sid] for sid in subject_ids])

skf_outer = StratifiedKFold(n_splits=5, shuffle=True, random_state=
    RANDOM_STATE)

for fold, (trainval_idx, test_idx) in enumerate(skf_outer.split(
    subject_ids, y)):

    test_sids     = [subject_ids[i] for i in test_idx]
    trainval_sids = [subject_ids[i] for i in trainval_idx]
    y_trainval    = np.array([labels_dict[sid] for sid in
        trainval_sids])

    # Single inner split: 3/4 train, 1/4 validation
    skf_inner = StratifiedKFold(n_splits=4, shuffle=True,
        random_state=RANDOM_STATE)
    inner_train_idx, inner_val_idx = next(skf_inner.split(
        trainval_sids, y_trainval))

    train_sids = [trainval_sids[i] for i in inner_train_idx]
    val_sids   = [trainval_sids[i] for i in inner_val_idx]

    # --- Data augmentation (train only) ---
    # Each training subject is tripled: original, Gaussian noise,
        time shift
    augmented_ts_data, augmented_labels = {}, {}
    for sid in train_sids:
        df, lab = ts_data[sid], labels_dict[sid]
        augmented_ts_data[f"{sid}_orig"]  = df
        augmented_ts_data[f"{sid}_noise"] = augment_add_noise(df)
        augmented_ts_data[f"{sid}_shift"] = augment_time_shift(df)
        augmented_labels[f"{sid}_orig"]   = lab
```

```
        augmented_labels[f"{sid}_noise"]  = lab
        augmented_labels[f"{sid}_shift"]  = lab

    # Val and test subjects are kept without augmentation
    for sid in val_sids + test_sids:
        augmented_ts_data[sid] = ts_data[sid]
        augmented_labels[sid]  = labels_dict[sid]

    # --- PLV threshold computed on train set only (no leakage) ---
    train_offdiag  = np.concatenate([
        plv_matrices[sid][np.triu_indices(plv_matrices[sid].shape[0], k=1)]
        for sid in train_sids
    ])
    fold_threshold = np.percentile(train_offdiag, PLV_PERCENTILE)

    # --- Feature and graph construction ---
    # Node features are enriched with two graph-topology descriptors:
    #   - degree    : number of edges per node
    #   - clustering: local clustering coefficient
    adj_matrices, combined_node_features = {}, {}
    for sid, df in augmented_ts_data.items():
        node_feats = np.vstack([extract_node_features(df.iloc[:, j].values)
                                for j in range(df.shape[1])])
        plv = generate_plv_matrix(df)
        adj = plv_to_adj(plv, method="global_threshold", threshold=fold_threshold)
        adj_matrices[sid] = adj

        G     = nx.from_numpy_array(adj)
        deg   = np.array([d for _, d in G.degree()])
        clust = np.array(list(nx.clustering(G).values()))

        # Concatenate spectral + topological features per node
        combined_node_features[sid] = np.concatenate(
            [node_feats, np.vstack([deg, clust]).T], axis=1
        )

    # --- Train / val / test dictionaries ---
    train_aug_sids = augmentation(train_sids)

    train_node_features = {sid: combined_node_features[sid] for sid in train_aug_sids}
    train_adj_d         = {sid: adj_matrices[sid]           for sid in train_aug_sids}
    train_labels_d      = {sid: augmented_labels[sid]       for sid in train_aug_sids}

    val_node_features = {sid: combined_node_features[sid] for sid in val_sids}
    val_adj_d         = {sid: adj_matrices[sid]           for sid in val_sids}
    val_labels_d      = {sid: augmented_labels[sid]       for sid in val_sids}
```

```python

    test_node_features = {sid: combined_node_features[sid] for sid in test_sids}
    test_adj_d         = {sid: adj_matrices[sid]           for sid in test_sids}
    test_labels_d      = {sid: augmented_labels[sid]       for sid in test_sids}

    # --- Training ---
    metrics, model, scaler, epoch_info, \
    y_true_val, y_pred_val, y_prob_val, histories = train_and_evaluate_gcn(
        train_node_features, train_adj_d, train_labels_d,
        val_node_features,   val_adj_d,   val_labels_d,
        N_EPOCHS, BATCH_SIZE, LR, CLASS_WEIGHT_MULTIPLIER,
        'precision_at_min_recall', 0.75
    )

    # --- Test evaluation ---
    test_data_list = build_gcn_data_list(
        list(test_node_features.keys()),
        test_node_features, test_adj_d, test_labels_d, scaler=scaler
    )
    test_loader = DataLoader(test_data_list, batch_size=BATCH_SIZE, shuffle=False)

    y_true, y_prob = [], []
    with torch.no_grad():
        for batch in test_loader:
            batch = batch.to(next(model.parameters()).device)
            probs = F.softmax(model(batch), dim=1)[:, 1].cpu().numpy()
            y_prob.extend(probs); y_true.extend(batch.y.cpu().numpy())

    # Threshold from val transferred to test (no leakage)
    best_th, _ = select_best_threshold_variant(y_true_val, y_prob_val, min_recall=0.75)
    y_pred = (np.array(y_prob) >= best_th).astype(int)

    test_metrics = {
        'accuracy':  accuracy_score(y_true, y_pred),
        'precision': precision_score(y_true, y_pred, zero_division=0),
        'recall':    recall_score(y_true, y_pred, zero_division=0),
        'f1':        f1_score(y_true, y_pred, zero_division=0),
        'roc_auc':   roc_auc_score(y_true, y_prob)
    }
    all_metrics.append(test_metrics)

# --- Final results aggregated across folds ---
avg_metrics = {k: np.mean([m[k] for m in all_metrics]) for k in all_metrics[0]}
std_metrics = {k: np.std([m[k] for m in all_metrics])  for k in all_metrics[0]}
```

```python

for k in avg_metrics:
    print(f"{k.upper():12}: {avg_metrics[k]:.4f} +/- {std_metrics[k
        ]:.4f}")
```
